# An Ecological Alternative to Snodgrass & Vanderwart: 360 High Quality Colour Images with Norms for Seven Psycholinguistic Variables

**DOI:** 10.1371/journal.pone.0037527

**Published:** 2012-05-25

**Authors:** Francisco Javier Moreno-Martínez, Pedro R. Montoro

**Affiliations:** Departamento de Psicología Básica I, Universidad Nacional de Educación a Distancia, Madrid, Spain; CSIC-Univ Miguel Hernandez, Spain

## Abstract

This work presents a new set of 360 high quality colour images belonging to 23 semantic subcategories. Two hundred and thirty-six Spanish speakers named the items and also provided data from seven relevant psycholinguistic variables: age of acquisition, familiarity, manipulability, name agreement, typicality and visual complexity. Furthermore, we also present lexical frequency data derived from Internet search hits. Apart from the high number of variables evaluated, knowing that it affects the processing of stimuli, this new set presents important advantages over other similar image corpi: (a) this corpus presents a broad number of subcategories and images; for example, this will permit researchers to select stimuli of appropriate difficulty as required, (e.g., to deal with problems derived from ceiling effects); (b) the fact of using coloured stimuli provides a more realistic, ecologically-valid, representation of real life objects. In sum, this set of stimuli provides a useful tool for research on visual object-and word- processing, both in neurological patients and in healthy controls.

## Introduction

Throughout the last 30 years, many clinical and experimental studies on cognitive processing (i.e., exploring memory, attention or language) have been performed with the items created by Snodgrass and Vanderwart (S&V) [1]. These authors standardized their stimuli in four variables relevant to cognitive processing: familiarity, image agreement, name agreement and visual complexity. Experimental control of these variables is essential because they are known to affect cognitive processing both of pictorial and verbal material. Thus, more familiar items, those with higher name and image agreement, as well as those with lesser visual complexity, are more easily named both by intact and neurological participants [2]–[5].

Apart from these variables, other cognitive and psycholinguistic variables such as age of acquisisiton (AoA) and manipulability and typicality of items significantly affect cognitive processing. Thus, AoA is a powerful predictor of object-naming performance both in normal and brain-injured individuals, with earlier acquired words being more easily processed than later acquired ones [6], [7]. Similarly, there is a significant relationship between the degree of manipulability of an object; that is, the degree of use of the human hand that is necessary for an object to perform its function and its semantic representation (e.g., [8]–[12]). Indeed, it has been proposed that differences in manipulability could explain category effects on object identification, consisting of a better performance with items from the domain of nonliving things (e.g., tools) compared to living things (e.g., animals; see [13], for a review). Lastly, typicality of items (i.e., how typical, or representative, a member is of a category) is another important psycholinguistic variable. Classic studies by Eleanor Rosch showed the relevance of this variable and its strong influence on performance in tasks assessing cognitive processing and memory, language use and communication, or development-related phenomena such as category learning and conceptual development (see, for example, [14]–[16]). Similarly, typicality of items has also been found to significantly impact the performance of neurological patients (e.g., aphasics: [17]). Despite the relevance of typicality in normal and damaged cognitive processing, most of the recent normative works and new semantic tests have not paid close attention to this variable (for example, [1], [18]–[28]; but see also [29]–[31]). Likewise, only a few recent works have provided ratings of AoA [19] or manipulability [10], and, to our knowledge, only [25], [30] have presented ratings of both variables concurrently, but with a relatively sparse number of items, as they only studied 140 [30] and 112 [25] coloured stimuli.

Some recent concerns respect to S&V corpi are related to the ecological validity of the stimuli and ceiling effects in the responses. Items from S&V consist of black and white line drawings. From an ecological view, the validity of studies using this type of stimuli has been questioned [28]. Colour is an essential attribute of objects and, except for unusual pathologies, it is difficult to separate colour from real world objects [32], [33]. Consequently, the number of works using coloured items, providing a more realistic representation of objects, as well as studies normalising coloured stimuli, have been progressively increasing (see, for example, [19]–[21], [23], [27], [28], [34]–[42]). Regarding ceiling effects, it has been observed that most of the items from S&V are easily named by healthy participants, at least under normal viewing conditions. This facilitates non-damaged participants showing ceiling effects in studies that involve the processing of objects, especially when using not very demanding tasks, (e.g., picture naming; see [37], [43]). As shown by Laws and collaborators in studies on category-specificity, this problem may distort both the degree and type of deficit reported in patients [37], [43].

The goal of the present work was twofold: (a) to present a broad set of high quality ecological colour photographs, on white backgrounds, across a difficulty range to deal with problems derived from ceiling effects; and (b) to give detailed norms, derived from a large group of healthy participants, of several relevant psycholinguistic variables, some of them not sufficiently studied in several previous works: AoA, familiarity, manipulability, name agreement, typicality and visual complexity, as well as lexical frequency. Furthermore, indexes of individual item analysis, including a measure of item difficulty and two indexes of item discrimination have been included.

## Methods

### Item selection

Following previous normative and semantic assessment studies, we selected 23 semantic subcategories (and their items) based on relevant theoretical and methodological reasons [1], [19]–[21], [23], [25]–[29], [30], [44], [45]. Consequently, we included problematic/atypical subcategories, such as body parts, musical instruments or foodstuff [13], [46], [47], different types of plant life subcategories [48]–[51]; insects [50]; subcategories differing in their degree of manipulability, such as buildings or tools [10]–[12]. As a result, we included ten subcategories from the living domain: animals, birds, body parts, dried fruits, insects, flowers, fruits, sea creatures, trees and vegetables; and twelve subcategories from the nonliving domain: buildings, clothing, foodstuff, furniture, jewellery, kitchen utensils, musical instruments, office material, sports/games, tools, vehicles and weapons; plus the subcategory of the nonliving natural things, such as a mountain or a stone. Table 1 contrasts the present work with previous normative studies carried out with coloured stimuli—plus the classic findings by S&V—regarding the number of categories and items studied.

**Table 1:** Present study compared to previous normative ones with coloured stimuli (Adlington et al., 2008; Brodeur et al., 2010; Moreno-Martínez et al., 2011; and Viggiano et al., 2004), plus the one by S&V, concerning the categories and number of items studied.

Present study		Adlington's	Brodeur's	M-Martínez's	S&V's	Viggiano's
**1. Animals**	**21**	08	---	10	30	42
**2. Birds**	**20**	08	---	---	08	---
**3. Body parts**	**20**	10	---	10	12	---
**4. Flowers**	**12**	08	---	10	---	---
**5. Fruits**	**21**	10	---	10	11	---
**6. Insects**	**17**	08	---	10	08	---
**7. Marine creatures**	**18**	---	---	---	---	---
**8. Nuts**	**11**	---	---	---	---	---
**9. Trees**	**11**	---	---	10	---	---
**10. Vegetables**	**20**	09	---	10	13	36
**11. Buildings**	**15**	10	---	10	---	---
**12. Clothing**	**13**	11	28	10	19	12
**13. Desk material**	**15**	---	38	---	---	---
**14. Food**	**15**	07	78	---	---	10
**15. Furniture**	**15**	12	02	10	14	13
**16. Jewellery**	**12**	---	08	---	---	---
**17. Kitchen utensiles**	**13**	12	60	10	14	12
**18. Musical instruments**	**16**	11	04	---	09	06
**19. Sports/Games**	**16**	---	41	---	18	---
**20. Tools**	**15**	12	37	10	12	29
**21. Vehicles**	**13**	11	---	10	10	09
**22. Weapons**	**15**	---	---	---	07	---
**23. Nature**	**16**	---	11	---	---	---

**Note**: --- = Category not studied.

Following the aforementioned procedure, 360 items were selected, and colour photographs were obtained for each one. All the photographs were directly taken by the first author and a collaborator (Sara Cañamón). Subsequently, the images were removed from their original backgrounds (except for the nonliving natural things) and placed on a plain white background; the mean dimension of the images was 265×223 pixels. Regarding the left-right orientation of each image, it was decided that, for each category susceptible to being oriented (i.e., animals, vehicles or tools), half of the items were left-facing and the other half right-facing.

**Figure 1:**
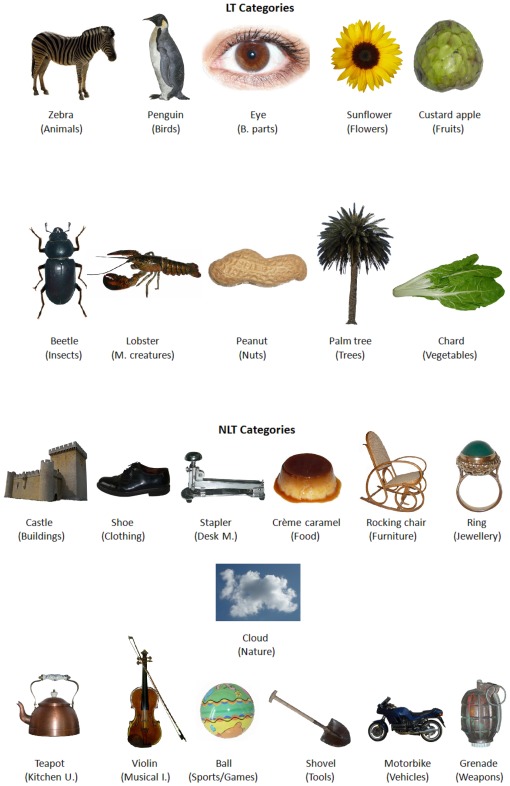
Several selected examples of the standardised stimuli (subcategory in brackets).

The experimental items were displayed to a sample of 236 participants (see Participants and Procedure sections) for naming the pictures and, then, for evaluating the five psycholinguistic variables included in the study: AoA, familiarity, manipulability, typicality and visual complexity. Several examples of items are presented in Figure 1; the whole set of items are included as supplemental material (Appendix S1).

### Participants

The sample consisted of 236 healthy Spanish-speaking undergraduate students (119 males; 117 females) with a mean age 36.7 years (*SD* = 10.9; range 19–63 years; Males *M* = 37.4, *SD* = 10.2; Females *M* = 36 *SD* = 11.5, F = 1.03, *n.s*.) and a mean number of years of education of 14.4 years (*SD* = 2.5; range 12–17 years; Males *M* = 14.6, *SD* = 2.5; Females *M* = 14.3, *SD* = 2.5, F = 1.3, *n.s*.). All had normal or corrected-to-normal vision, and Spanish was their first language. Any person with a known history of neurological disease, head trauma, or stroke was excluded. The student participants were assigned course credit for their participation in the study. The study was approved by the Bioethics Committee from the UNED and conforms with the Declaration of Helsinki. All participants provided written informed consent (approved by the Bioethics Committee from the UNED) for the collection of data and subsequent analysis. Additionally, participants were explained that they were free to suspend their participation in the experiments at any time and for any cause.

### Procedure

The 360 images were divided into three groups of items (120 each), namely lists A, B and C. We implemented a pseudorandom selection in order to ensure that the three resulting lists included a similar number of exemplars belonging to the 23 subcategories. The 236 participants were randomly assigned to work with one of the groups of items. Each group of items was evaluated by *n* = 77 (38 males; 39 females, list A), *n* = 80 (41 males; 39 females, list B), and *n* = 79 (40 males; 39 females, list C). Participants were tested individually in two sessions. They all carried out the naming session first and, subsequently, they rated the items for familiarity, age of acquisition, visual complexity, manipulability and typicality. The whole experiment, combined across both sessions, lasted approximately ninety minutes, with self-administered rest periods during the two sessions and between sessions. Each experimental session was preceded by the instructions provided by researchers and a practice phase to enable each participant to become familiar with the task, and, additionally, to generate the acquisition of anchor points for the stimulus ratings. In the practice phase, each participant observed ten pictures that were not included in the main stimulus set. The pictures were displayed on 19-inch LCD colour monitors with a screen resolution of 1024×768 pixels and a 32-bit color mode controlled by microcomputers running E-Prime 1.2 software (Psychology Software Tools, 1996–2002). Every monitor was calibrated by means of the Display Color Calibration tool available in Windows 7 Professional operating system (Microsoft corporation, 2009) including brightness, contrast, color balance and Gamma adjustments. Previously to the beginning of each experimental session, at least 45 minutes were provided to warm up the monitors. Periodically, the screens were carefully cleaned in order to ensure an optimal picture quality. Viewing distance was approximately 60 cm.

During the test phase, the 120 images were presented in a random order. Each image was preceded by a cross (+) for 500 ms, and remained on the screen for 3,000 msec (naming task phase) or until the participant responded (during the item rating phase). During the latter part of the task, visual complexity and typicality were always the first and the last variables evaluated, respectively; the rest of the variables were randomly displayed. To evaluate visual complexity, participants were asked to “rate the visual complexity of the image itself, rather than that of the object it represents”. To evaluate the remaining variables (AoA, familiarity, manipulability and typicality), participants were asked to “rate the object represented rather than the image itself”. When the participants evaluated the variables AoA, familiarity, manipulability and typicality, experimenters provided them with the canonical name of the item (i.e., the intended one). Additionally, when participants evaluated the typicality of the items, they were also provided with the category of the item on the screen (e.g., “animals” —category— for “elephant”—item).

#### Naming task

Participants were asked to name each image by typing its name with the keyboard on the screen. They were told to give the specific—rather than the general—name for the different items. For example, in the case of the subcategory of “trees”, if the participant knew the name of the item, he/she should give the name of that particular tree, e.g., “pine tree”, instead of the general name of “tree”. Participants were asked to type the initials for “don't know” (NC = “No Conozco”, in Spanish), if the image was unknown to them, to type “tip of the tongue” (PL = “Punta de la Lengua”, in Spanish) if they were momentarily unable to remember the name, or to type “don't remember” (NR = “No Recuerdo”, in Spanish). All their responses were automatically saved by the program. According to this task, “name agreement” was calculated based on the percentage of participants who named the item according to its canonical name.

#### AoA

Participants were asked to estimate the age in years at which they had learned each word following the same procedure of other similar previous studies (e.g., [4], [7]). Scores were obtained by asking participants to rate age of acquisition for each word on a seven-interval scale (range: 1 = 0–2 years; 7 = 13 years or more; see [25]).

#### Familiarity

Participants were instructed to rate each item, assessing “how usual or unusual the concept is in your realm of experience” on the basis of “how frequently you think about the concept, and how frequently you come into contact with the concept —both directly (e.g., seeing a real-life exemplar) and in a mediated way (e.g., represented in the media)”. Participants provided their responses on a 5-point Likert scale (1* = very unfamiliar*, 5* = very familiar*) by pressing the corresponding number on the keyboard.

#### Manipulability

Participants were instructed to rate each item, assessing “the degree to which using a human hand is necessary for this object to perform its function”. Participants provided their responses on a 5-point Likert scale (1* = never necessary*, 5* = totally indispensable*) by pressing the corresponding number on the keyboard.

*Typicality*: This reflects the degree to which a concept is a representative exemplar of its category. Scores were obtained by asking participants to rate on a 5-point scale (1 = *not at all prototypical*, 5 = *very prototypical*) how representative of its category they thought an exemplar was (e.g., *car* for *vehicles*).

#### Visual Complexity

Instructions from S&W's study were adapted to evaluate the visual complexity of the items. Consequently, participants were asked to evaluate “the amount of detail, intricacy of lines, pattern and quantity of colours presented in the image”. Participants recorded their responses on a 5-point scale (1* = very simple*, 5* = very complex*) by pressing corresponding numbers on the keyboard.

#### Lexical frequency

Owing to the unavailability of norms for all of the words in a standard Spanish corpus (e.g. [52]), we gathered norms for lexical frequency using an Internet search engine. This method is a viable alternative to the currently available databases and may even provide a more representative [53] as well as a constantly updating measure of word frequency [19] that has high convergent validity with other more classical databases. Furthermore, search engines permit the gathering of word frequency values for more unusual items that do not typically feature in conventional databases (see [19], [30], [54], [55]). With more than 250 million web pages, the AltaVista search engine (www.altavista.com) is one of the largest search engines currently available and, for this reason, it was selected for this process. These names were entered into the search function of AltaVista, and a search was performed, specifying that results should be for Spain and in Spanish only. The number of hits returned, after conversion to their natural logarithm, served as the frequency estimate for each word [19], [31], [53], [56].

## Results

### 1. Descriptive results

A summary of the rating data for each item is reported in Appendix S2 of the supplemental material. For each item, the following information is presented: 1) the number of order of each item; 2) the most frequent name in English and Spanish; 3) two measures of name agreement: the statistic *H* and the percentage of participants who produced the canonical/dominant name, plus the percentage of participants who produced the modal name of the item in those cases in which the latter did not match the dominant name. Although both indexes are measures of name agreement (statistic *H* and the percentage), the latter indicates only how dominant the most common name is in a sample, whereas H (or entropy [57]) is sensitive to how widely distributed responses are over all the unique names that are provided for a picture. Consequently, index *H* is more informative than name agreement (e.g., it gives information about the dispersion of the names). *H* was calculated according to the following formula:
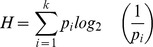
where k is the number of unique names given for a picture, and p_i_ is the proportion of the sample providing each unique name. *H* = 0 when there is perfect agreement among participants (e.g., just one name) and increases as agreement decreases. “Don't know”, “tip of the tongue” and “don't remember” responses were not taken into account to calculate index *H;* 4) the means and standard deviation for AoA, familiarity, manipulability, typicality, visual complexity and lexical frequency values expressed as a natural logarithm. Appendix S3 reports alternative names of each item; indexes of individual item analysis, including a measure of item difficulty and two indexes of item discrimination based on item-test correlations—point-biserial and biserial— are also included in Appendix S4. Table 2 presents summary statistics for all the aforementioned variables. Likewise, Table 3 shows summary statistics for all the variables for all the subcategories. Lastly, Table 4 shows Pearson correlations among the variables. In general, as with other normative studies, the standard psycholinguistic variables tend to correlate with each other (see [1], [19], [58], [59]).

**Table 2:** Summary statistics for all the variables.

	AoA	Fam	LF (Log)	Man	Typ	VC	NA	*H*
***M***	3.63	3.56	15.560	3.11	3.65	2.55	0.72	0.94
***SD***	1.30	0.88	14.190	1.26	0.86	0.74	0.28	0.87
**Median**	3.44	3.55	15.520	3.52	3.74	2.46	0.82	0.70
**Mode**	3.05	4.78	14.190	1.22	3.37	1.53	1.00	0.00
**Skew**	0.37	−0.25	−0.019	−0.30	−0.54	0.21	−0.86	0.83
**Kurtosis**	−0.73	−0.79	0.380	−1.43	−0.52	−0.77	−0.47	−0.21
**Range**	5.36	3.65	11.300	3.87	3.49	3.31	0.99	3.73
**Min**	1.37	1.32	9.010	1.01	1.46	1.18	0.01	0.00
**Max**	6.73	4.97	20.310	4.88	4.95	4.49	1.00	3.73
**Q1**	2.59	2.87	14.310	1.70	3.06	1.94	0.53	0.20
**Q3**	4.58	4.36	16.630	4.21	4.42	3.14	0.96	1.57

**Note**: AoA = Age of acquisision; Fam = Familiarity, LF = Lexical frequency (natural logarithm); Man = Manipulability; Typ = Typicality; VC = Visual complexity; % NA = Percentage of name agreement.

**Table 3:** Summary statistics for all the variables for each category.

	AoA		Fam		Man		Typicality		VC		LF (Log)		%NA	
	*M*	*SD*	*M*	*SD*	*M*	*SD*	*M*	*SD*	*M*	*SD*	*M*	*SD*	*M*	*SD*
**1. Animals**	3.46	1.39	2.91	0.74	1.42	0.41	3.60	0.87	3.19	0.36	15.22	1.63	0.79	0.25
**2. Birds**	3.88	1.21	3.01	0.75	1.45	0.28	3.47	0.67	3.25	0.44	15.16	1.10	0.66	0.25
**3. Body parts**	2.74	1.50	4.13	0.92	2.09	0.82	4.07	0.70	2.36	0.53	16.80	1.56	0.74	0.29
**4. Flowers**	4.18	1.15	3.43	0.67	2.06	0.25	3.82	0.69	2.73	0.50	15.56	1.80	0.52	0.31
**5. Fruits**	3.50	1.46	3.92	0.83	3.71	0.21	3.75	0.93	1.79	0.39	15.78	1.44	0.77	0.26
**6. Insects**	3.28	1.04	3.36	0.80	1.41	0.18	3.87	0.67	2.99	0.48	14.76	1.14	0.75	0.20
**7. Marine creatures**	3.12	0.99	3.86	0.54	4.26	0.56	3.63	1.67	2.63	0.74	15.67	1.67	0.89	0.12
**8. Nuts**	3.57	0.72	3.70	0.54	3.67	0.47	3.85	0.70	1.96	0.31	15.10	1.68	0.67	0.25
**9. Trees**	4.40	.81	3.28	0.53	1.71	0.24	3.71	0.49	2.82	0.43	15.29	0.96	0.37	0.32
**10. Vegetables**	3.84	1.14	3.93	0.74	3.70	0.27	3.84	0.58	1.97	0.49	15.17	0.94	0.74	0.27
**11. Buildings**	4.01	1.44	3.18	0.89	2.66	0.50	2.81	1.07	3.35	0.73	16.65	2.14	0.70	0.24
**12. Clothing**	2.85	1.33	4.30	0.88	3.81	0.48	3.86	1.17	1.89	0.39	15.43	1.46	0.89	0.18
**13. Desk material**	3.47	1.14	4.25	0.52	4.55	0.20	4.09	0.57	1.83	0.53	15.48	1.74	0.77	0.29
**14. Food**	3.48	1.35	3.98	0.92	4.03	0.34	3.47	0.90	2.17	0.79	14.54	2.04	0.74	0.27
**15. Furniture**	3.30	1.47	4.23	0.68	3.19	0.54	3.89	0.88	2.45	0.48	15.97	1.72	0.79	0.21
**16. Jewellery**	4.03	1.12	3.39	0.61	3.88	0.44	3.77	0.89	2.79	0.50	15.97	1.73	0.66	0.23
**17. Kitchen utensiles**	3.88	1.43	3.84	1.01	4.41	0.30	3.68	1.07	2.20	0.64	14.54	1.33	0.63	0.29
**18. Musical instuments**	4.05	1.24	3.09	0.70	4.69	0.13	3.79	0.79	3.13	0.73	15.05	1.74	0.77	0.31
**19. Sports/Games**	3.12	0.99	3.86	0.54	4.26	0.56	3.63	0.63	2.63	0.73	15.67	1.67	0.89	0.12
**20. Tools**	4.21	1.20	3.49	0.73	4.53	0.18	3.82	0.69	1.93	0.39	14.74	2.07	0.70	0.28
**21. Vehicles**	2.93	0.97	3.78	.65	3.93	0.55	3.32	1.18	2.98	0.62	16.83	1.83	0.83	0.15
**22. Weapons**	3.91	1.01	2.59	.49	4.20	0.52	3.19	1.02	2.56	0.76	15.80	1.89	0.78	0.25
**23. Nature**	2.85	1.01	3.71	.79	1.49	0.59	3.66	0.73	2.68	0.72	17.50	1.53	0.67	0.29

Note: AoA = Age of acquisision; Fam = Familiarity, LF = Lexical frequency (natural logarithm); Man = Manipulability; VC = Visual complexity; % NA = Percentage of name agreement.

**Table 4:** Correlation matrix for naming performance and psycholinguistic variables.

	AoA	Fam	LF	Man	Typ	VC	%NA	*H*
AoA	1	−.82*	−.57*	−.05	−.72*	.34*	−.68*	.67*
Fam		1	.47*	.29*	.75*	−.57*	.63*	−.61*
LF			1	−.05	.45*	−.10	.42*	−.40
Man				1	.10	−.36*	.19*	−.20*
Typ					1	−.27*	.52*	−.51*
VC						1	−.25*	.26*
NA							1	−.93*
*H*								1

**Note**: AoA = Age of acquisision; Fam = Familiarity, LF = Lexical frequency; Man = Manipulability; Typ = Typicality; VC = Visual complexity; % NA = Percentage of name agreement.

* *p*<.01.

### 2. Reliability and validity of the study

To establish validity, we compared our norms/stimuli with those of the classical S&V, collected in USA, plus four recent studies which, like ours, were conducted with high quality colour images and coloured pictures, collected in United Kingdom, Canada, Italy and Spain, respectively: [1], [19], [20], [28], [30]. Pearson's correlations, including those items sharing the same name in the four studies (n = 50 with [19], n = 68 with [20], n = 113 with [30], n = 80 with [28], and n = 106 with [1]) are shown in Table 5. A high pattern of significant correlations (fluctuating between .25 and .99) was found among the diverse variables observed across the five studies. So, even where compared across English (different countries, languages and studies), Italian and Spanish, the ratings remain highly correlated. Regarding reliability, Cronbach's alpha coefficients were also high: α = .83 (name agreement), α = .97 (visual complexity, familiarity and manipulability) and α = .98 (AoA and typicality).

**Table 5:** Correlations between current stimuli and those of Adlington et al. (2009), Brodeur et al. (2010), Moreno-Martínez et al. (2011), Snodgrass and Vanderwart (1980), and Viggiano et al. (2004)^1^.

	Items (n)	AoA	Fam	LF	Man	VC	%NA
**Adlington et al.'s**	50	.84	.78	.74	n.e.	.66	.62
**Brodeur et al.'s**	68	n.e.	.76	n.e.	.48	.68	.25
**Moreno-Martínez et al.'s**	113	.99	.98	.99	.88	.92	.94
**S&V**	106	.81^2^	.79	.62	n.e.	.76	.41
**Viggiano et al.'s (English)**	80	n.e.	.73	n.e.	n.e.	.80	.34
**Viggiano et al.'s (Italian)**	80	n.e.	.77	n.e.	n.e.	.84	.46

**Note**: AoA = Age of acquisision; Fam = Familiarity, LF = Lexical frequency; VC = Visual complexity; % NA = Percentage of name agreement, n.e. = not evaluated.

1 = Viggiano et al.'s work studied two samples: English and Italian speakers evaluated the same items.

2 = 39 items (in their original study, S&V presented AoA data only for some items).

## Discussion

The goal of the present work was twofold: (a) to present a broad set of high quality ecological colour photographs across a range of difficulty, to deal with problems derived from ceiling effects; and (b) to give detailed norms, derived from a group of healthy participants, of several relevant psycholinguistic variables. To the best of our knowledge, this work is the first to provide such a high number of quality ecological items (360), pertaining to so many different (23) subcategories and providing indexes of seven relevant psycholinguistics variables: age of acquisition, familiarity, lexical frequency, manipulability, name agreement, typicality and visual complexity, gathered from such a large number of participants (n = 236). Another main contribution of our study, compared to previous recent normative works, is that it incorporates item analyses, for those authors interested in selecting the more suitable items according to their goals and recently collected norms on typicality.

Recent normative works have provided valuable data from a high number of coloured items and have also presented ratings for relevant psycholinguistics variables [19], [20], [30], [28]. However, (i) they have excluded theoretically relevant subcategories, such as animals, body parts, buildings and vehicles ([20], also did not evaluate typicality), (ii) they have provided ratings for only several psycholinguistics variables: familiarity, name agreement and visual complexity ([28], did not include body parts) or (iii) the number of items evaluated is relatively sparse, compared to the 260 items originally studied by S&V (147: [19]; 140: [30]; 174: [28]). The category-specific literature has convincingly shown that there are important differences within the living domain (e.g., animals and plant life—fruits, flowers and vegetables) between the animals and plant-life subcategories [13]. Similarly, processing differences have been reported within the non-living domain (e.g., tools, vehicles and furniture), between small manipulable objects, such as tools, and large outdoor objects, such as buildings [12]. In their domain-specific theory, Caramazza and collaborators posited that, for the subcategories of items for which rapid identification confers reproductive advantages, natural selection has produced specialized, dissociable neural pathways—modules [48], [60], [61]. According to these proposals, such modules exist for animals and plant life, although the domains of tools and conspecifics have recently been incorporated into this view [62]. Similarly, within the nonliving thing domain, Warrington and McCarthy [12] reported a case that revealed a clear dissociation within this domain: a greater impairment in identifying small and manipulable objects, compared to large and non-manipulable things. Regarding psycholinguistics variables, while familiarity [3], name agreement [4] and visual complexity [5] have been shown to be significantly relevant to the processing of pictorial and verbal material, both in control participants and patients, other no less relevant variables, such as age of acquisition [2], manipulability [63], typicality [17] and word frequency [64] have also robustly revealed their impact on normal and impaired processing of items. Consequently, works providing normative data for these variables are, in our view, particularly demanded in the object processing arena. Likewise, the above commented semantic specialization (i.e., differences between manipulable and non-manipulable things/biologically derived modules), strongly recommend having a sufficient number of items that make it possible to elucidate these theoretical issues.

Validity indexes showed that our stimuli had similar features as those of other corpi and they presente high internal consistency as well; this suggests that the new corpus has adequate psychometric characteristics. Likewise, the fact that our scales presented high cross-language correlations with similar studies indicates that our stimuli are suitable to be used in countries other than Spain and in different cultures with different languages.

Although we have attempted to address the methodological issues of this literature reviewed in the Introduction, there remains one limitation in the current study: the fact that cognitive/conceptual effects are able to drive the categorization beyond the low level features. As mentioned in Introduction, we selected our categories —and stimuli— in a “top-down perspective”, based on relevant theoretical reasons, and mainly derived from Cognitive Neuropsychology arena [1], [10]–[13], [19]–[21], [23], [25]–[30], [44], [45], [47]. From a different perspective, vision studies from psychophysical and neurophysiological field have, traditionaly, made used of accurate low-level quantitative methods to define the physical parameters of naturalistic photographs, in order to explore basic aspects of the human visual system (see, e.g., [65]–[67]). However, it should be recognized that the human visual system is sufficiently adaptable to make possible that different low level features in the stimuli can be compensated to obtain higher level invariant categorizations. Clearly, this is something that cannot be taken for granted and should be recognized in any study dealing with pictorial stimuli.

Beyond the low level properties of objects, another relevant point has been relatively ignored in the previous literature on normative and semantic assessment studies. This point is related to the control of the relations between objects specified by abstract feature spaces (see [68], for a review). Most of the recently developed corpi have been designed according to arbitrary criteria for the selection of the categories and the assigning of their stimuli. In contrast, an alternative selection method could take advantage of the semantic structural descriptions derived from hierarchical Bayesian models, which fits quite well the human performance in semantic induction tasks [68], [69]. This procedure should be seriously considered by researchers in order to develop more accurate instruments in this field.

To conclude, the present work provides a useful tool for researchers examining language, memory, object- and word-processing, particularly for those interested in comparing healthy versus neurologically damaged populations. Accordingly, the new instrument, in combination with other recently developed corpi, is intended to be an ecological alternative to the corpus developed by Snodgrass and Vanderwart thirty years ago, especially, but not exclusively, in a Spanish-speaking population.

## Supporting Information

Appendix S1 **Colour photographs of the 360 items.**
(RAR)

Appendix S2 **Normative psycholinguistic ratings for each item.**
(DOC)

Appendix S3 **Proportion (in brackets) of target names, alternative names, acceptable synonyms of each item, plus “Don't know” (DK), “Don't remember” (DR), and “Tip of the tongue” (TOT) responses.**
(DOC)

Appendix S4 **Indexes of individual item analysis including a measure of item difficulty and two indexes of item discrimination based on item-test correlations (point-biserial and biserial).**
(DOC)
