# Supplementary material for: An Ecological Alternative to Snodgrass & Vanderwart: 360 High Quality Colour Images with Norms for Seven Psycholinguistic Variables
Source: PLoS One. 2012 May 25;7(5):e37527. doi: 10.1371/journal.pone.0037527 (PMC3360784; doi:10.1371/journal.pone.0037527)
Supplement: Appendix S2 — Normative psycholinguistic ratings for each item. (DOC) [file pone.0037527.s002.doc]

| **Number of order and Item** | | AoA | | Fam | | Man | | Typicality | | VC | | NADominant | | NA Modal | LF |
| --- | --- | --- | --- | --- | --- | --- | --- | --- | --- | --- | --- | --- | --- | --- | --- |
| **English** | **Spanish** | *M* | *SD* | *M* | *SD* | *M* | *SD* | *M* | *SD* | *M* | *SD* | *p* | *H*** | *p* | *Log* |
| ***1. ANIMALS*** |  |  |  |  |  |  |  |  |  |  |  |  |  |  |  |
| 1.1. Armadillo | Armadillo | 6.03 | 1.22 | 1.86 | 1.03 | 1.29 | 0.64 | 2.10 | 1.34 | 3.62 | 1.09 | .57 | 1.14 | .57 | 13.80 |
| 1.2. Bat | Murciélago | 3.39 | 0.93 | 2.95 | 1.26 | 1.20 | 0.59 | 2.87 | 1.18 | 3.41 | 1.13 | .96 | 0.10 | .96 | 14.87 |
| 1.3. Cat | Gato | 1.58 | 0.67 | 4.29 | 1.06 | 1.95 | 1.19 | 4.86 | 0.38 | 3.13 | 1.21 | 1.00 | 0.00 | 1.00 | 17.78 |
| 1.4. Cheetah | Guepardo | 4.85 | 1.55 | 2.23 | 1.07 | 1.27 | 0.65 | 3.15 | 1.19 | 3.48 | 1.12 | .32 | 2.57 | .32 | 13.11 |
| 1.5. Cow | Vaca | 2.00 | 0.97 | 4.10 | 1.01 | 2.59 | 1.33 | 4.73 | 0.55 | 2.55 | 1.27 | 1.00 | 0.00 | 1.00 | 17.00 |
| 1.6. Crocodile | Cocodrilo | 2.96 | 1.08 | 2.73 | 1.40 | 1.18 | 0.62 | 3.77 | 1.02 | 3.38 | 1.20 | .76 | 0.92 | .76 | 15.46 |
| 1.7. Dromedary | Dromedario | 4.34 | 1.65 | 2.48 | 1.24 | 1.66 | 1.06 | 3.19 | 1.23 | 2.99 | 1.17 | .50 | 1.00 | .50 | 12.45 |
| 1.8. Elephant | Elefante | 2.35 | 0.80 | 3.29 | 1.32 | 1.30 | 0.76 | 4.46 | 0.81 | 3.06 | 1.32 | 1.00 | 0.00 | 1.00 | 16.15 |
| 1.9. Giraffe | Jirafa | 2.49 | 1.04 | 3.41 | 1.41 | 1.25 | 0.74 | 4.18 | 0.84 | 3.24 | 1.18 | .97 | 0.10 | .97 | 14.39 |
| 1.10. Hippopotamus | Hipopótamo | 3.00 | 0.99 | 2.86 | 1.22 | 1.27 | 0.76 | 3.59 | 1.04 | 2.33 | 1.02 | .82 | 0.76 | .82 | 13.81 |
| 1.11. Horse | Caballo | 2.00 | 0.77 | 3.85 | 1.08 | 2.44 | 1.36 | 4.68 | 0.71 | 3.34 | 1.33 | .99 | 0.11 | .99 | 17.53 |
| 1.12. Kangaroo | Canguro | 3.37 | 1.25 | 2.67 | 1.33 | 1.16 | 0.41 | 3.37 | 1.16 | 3.13 | 1.02 | 1.00 | 0.00 | 1.00 | 15.50 |
| 1.13. Lioness | Leona | 2.72 | 1.07 | 3.28 | 1.36 | 1.27 | 0.78 | 4.44 | 0.76 | 3.20 | 1.18 | .59 | 1.27 | .59 | 16.86 |
| 1.14. Lynx | Lince | 4.80 | 1.46 | 2.52 | 1.05 | 1.29 | 0.64 | 3.29 | 1.20 | 3.65 | 1.13 | .74 | 1.03 | .74 | 15.67 |
| 1.15. Platypus | Ornitorrinco | 5.76 | 1.42 | 1.76 | 0.96 | 1.13 | 0.44 | 2.21 | 1.29 | 3.72 | 0.98 | .46 | 1.20 | .46 | 13.59 |
| 1.16. Rhino | Rinoceronte | 3.59 | 1.29 | 2.63 | 1.25 | 1.15 | 0.43 | 3.49 | 1.11 | 3.32 | 1.18 | .96 | 0.10 | .96 | 13.90 |
| 1.17. Snake | Serpiente | 2.52 | 0.83 | 2.95 | 1.27 | 1.34 | 0.75 | 3.46 | 1.01 | 2.48 | 1.07 | .92 | 0.34 | .92 | 16.12 |
| 1.18. Tapir | Tapir | 6.48 | 1.06 | 1.35 | 0.68 | 1.20 | 0.52 | 1.86 | 1.25 | 3.35 | 0.85 | .18 | 2.37 | .29 | 12.60 |
| 1.19. Tiger | Tigre | 2.59 | 1.23 | 3.35 | 1.33 | 1.22 | 0.47 | 4.53 | 0.75 | 3.35 | 1.38 | .99 | 0.10 | .99 | 17.28 |
| 1.20. Turtle | Tortuga | 2.66 | 1.04 | 3.27 | 1.24 | 1.35 | 0.79 | 3.68 | 1.04 | 3.19 | 1.09 | .90 | 0.46 | .90 | 16.13 |
| 1.21. Zebra | Cebra | 3.15 | 1.25 | 3.21 | 1.25 | 1.29 | 0.65 | 3.77 | 1.04 | 3.17 | 1.36 | .99 | 0.00 | .99 | 15.64 |
| ***2. BIRDS*** |  |  |  |  |  |  |  |  |  |  |  |  |  |  |  |
| 2.1. Barn owl | Lechuza | 4.09 | 1.64 | 2.62 | 1.09 | 1.22 | 0.61 | 3.30 | 1.15 | 3.29 | 1.20 | .47 | 1.34 | .47 | 14.83 |
| 2.2. Duck | Pato | 2.03 | 0.85 | 3.99 | 0.96 | 1.71 | 1.09 | 4.18 | 0.99 | 3.49 | 1.21 | .93 | 0.44 | .93 | 16.82 |
| 2.3. Goldfinch | Jilguero | 4.59 | 1.63 | 2.81 | 1.23 | 1.36 | 0.79 | 3.56 | 1.06 | 3.63 | 1.11 | .34 | 2.25 | .34 | 13.80 |
| 2.4. Goose | Oca | 3.28 | 1.54 | 3.28 | 1.10 | 1.70 | 1.04 | 3.49 | 0.89 | 2.38 | 0.92 | .52 | 1.59 | .52 | 16.05 |
| 2.5. Hen | Gallina | 2.01 | 0.76 | 4.11 | 1.06 | 2.28 | 1.25 | 4.06 | 1.08 | 3.00 | 1.30 | .87 | 0.55 | .87 | 16.30 |
| 2.6. Hummingbird | Colibrí | 5.16 | 1.48 | 2.54 | 1.23 | 1.22 | 0.65 | 3.13 | 1.22 | 4.22 | 1.02 | .71 | 0.95 | .71 | 14.32 |
| 2.7. Kiwi | Kiwi | 6.42 | 1.14 | 1.49 | 0.90 | 1.33 | 0.75 | 1.80 | 1.15 | 3.41 | 1.01 | .32 | 2.47 | .32 | 15.05 |
| 2.8. Magpie | Urraca | 4.55 | 1.54 | 2.72 | 1.37 | 1.26 | 0.65 | 3.29 | 1.22 | 3.38 | 0.93 | .21 | 2.28 | .21 | 14.21 |
| 2.9. Ostrich | Avestruz | 3.54 | 1.32 | 2.91 | 1.10 | 1.35 | 0.70 | 3.32 | 1.05 | 3.18 | 1.13 | .96 | 0.20 | .96 | 14.54 |
| 2.10. Owl | Búho | 3.60 | 1.46 | 3.03 | 1.15 | 1.35 | 0.80 | 3.76 | 1.08 | 3.37 | 1.15 | .81 | 0.70 | .81 | 14.79 |
| 2.11. Partridge | Perdiz | 4.24 | 1.50 | 2.86 | 1.25 | 1.77 | 1.17 | 3.65 | 1.00 | 3.54 | 1.19 | .51 | 1.74 | .51 | 15.17 |
| 2.12. Pelican | Pelícano | 4.56 | 1.47 | 2.38 | 1.15 | 1.22 | 0.55 | 2.99 | 1.15 | 3.63 | 1.19 | .70 | 1.06 | .70 | 13.62 |
| 2.13. Penguin | Pingüino | 3.30 | 1.10 | 2.77 | 1.25 | 1.22 | 0.57 | 2.57 | 1.16 | 3.09 | 1.12 | .99 | 0.00 | .99 | 15.05 |
| 2.14. Pheasant | Faisán | 5.63 | 1.44 | 1.91 | 0.88 | 1.42 | 0.87 | 2.65 | 1.23 | 3.78 | 1.01 | .26 | 2.59 | .26 | 15.61 |
| 2.15. Pigeon | Paloma | 2.25 | 0.95 | 4.47 | 0.80 | 1.52 | 0.89 | 4.58 | 0.67 | 2.70 | 1.07 | .99 | 0.00 | .99 | 16.91 |
| 2.16. Raven | Cuervo | 3.81 | 1.19 | 3.14 | 1.13 | 1.35 | 0.79 | 3.71 | 1.03 | 2.39 | 0.98 | .77 | 1.04 | .77 | 16.37 |
| 2.17. Rooster | Gallo | 2.33 | 0.83 | 3.81 | 1.10 | 1.86 | 1.20 | 4.06 | 0.97 | 3.10 | 1.19 | .90 | 0.46 | .90 | 16.92 |
| 2.18. Seagull | Gaviota | 3.61 | 1.30 | 3.30 | 1.21 | 1.27 | 0.65 | 3.91 | 0.94 | 2.91 | 1.13 | .83 | 0.57 | .83 | 15.34 |
| 2.19. Sparrow | Gorrión | 3.27 | 1.38 | 3.84 | 1.17 | 1.29 | 0.70 | 4.39 | 0.77 | 3.15 | 1.25 | .50 | 1.83 | .50 | 14.21 |
| 2.20. Toucan | Tucán | 5.30 | 1.43 | 2.30 | 1.07 | 1.30 | 0.70 | 3.06 | 1.23 | 3.41 | 0.91 | .58 | 1.83 | .58 | 13.40 |
| ***3. BODY PARTS*** |  |  |  |  |  |  |  |  |  |  |  |  |  |  |  |
| 3.1. Arm | Brazo | 1.62 | 0.83 | 4.87 | 0.47 | 2.71 | 1.60 | 4.73 | 0.70 | 1.73 | 0.83 | .96 | 0.17 | .96 | 17.27 |
| 3.2. Beard | Barba | 2.62 | 1.24 | 3.32 | 1.49 | 2.49 | 1.32 | 2.51 | 1.27 | 3.10 | 1.35 | .43 | 0.85 | .43 | 16.43 |
| 3.3. Bone | Hueso | 2.61 | 0.88 | 4.08 | 1.15 | 1.65 | 1.03 | 4.13 | 1.07 | 1.43 | 0.57 | 1.00 | 0.00 | 1.00 | 16.61 |
| 3.4. Brain | Cerebro | 4.14 | 1.43 | 4.09 | 1.08 | 1.49 | 0.82 | 3.87 | 1.07 | 2.86 | 1.40 | .86 | 0.61 | .86 | 17.56 |
| 3.5. Ear | Oreja | 1.54 | 0.72 | 4.77 | 0.62 | 1.91 | 1.14 | 4.64 | 0.72 | 2.31 | 1.19 | .99 | 0.10 | .99 | 16.97 |
| 3.6. Eye | Ojo | 1.53 | 0.77 | 4.87 | 0.47 | 1.78 | 1.20 | 4.69 | 0.57 | 2.71 | 1.36 | 1.00 | 0.00 | 1.00 | 18.37 |
| 3.7. Finger | Dedo | 1.47 | 0.68 | 4.91 | 0.36 | 3.78 | 1.69 | 4.43 | 1.01 | 1.95 | 0.93 | .90 | 0.56 | .90 | 17.18 |
| 3.8. Foot | Pie | 1.49 | 0.72 | 4.92 | 0.31 | 2.19 | 1.23 | 4.81 | 0.56 | 2.22 | 1.15 | 1.00 | 0.00 | 1.00 | 18.93 |
| 3.9. Hand | Mano | 1.37 | 0.51 | 4.94 | 0.29 | 3.99 | 1.53 | 4.95 | 0.27 | 2.70 | 1.33 | 1.00 | 0.00 | 1.00 | 19.61 |
| 3.10. Kidney | Riñón | 4.33 | 1.33 | 3.01 | 1.27 | 1.70 | 1.09 | 3.63 | 1.19 | 1.86 | 1.14 | .47 | 1.98 | .47 | 15.86 |
| 3.11. Leg | Pierna | 1.56 | 0.71 | 4.92 | 0.27 | 2.03 | 1.24 | 4.81 | 0.56 | 2.01 | 1.06 | 1.00 | 0.00 | 1.00 | 16.63 |
| 3.12. Liver | Hígado | 4.62 | 1.42 | 2.95 | 1.34 | 1.42 | 0.78 | 3.86 | 1.13 | 2.43 | 1.22 | .68 | 1.25 | .68 | 16.13 |
| 3.13. Lung | Pulmón | 4.25 | 1.48 | 2.68 | 1.34 | 1.37 | 0.88 | 3.73 | 1.14 | 2.62 | 1.15 | .42 | 2.18 | .42 | 15.97 |
| 3.14. Mouth | Boca | 1.51 | 0.66 | 4.89 | 0.45 | 2.01 | 1.25 | 4.53 | 0.84 | 1.92 | 0.78 | .24 | 0.80 | .76 | 18.64 |
| 3.15. Nose | Nariz | 1.54 | 0.72 | 4.85 | 0.54 | 2.09 | 1.21 | 4.62 | 0.79 | 1.68 | 0.88 | 1.00 | 0.00 | 1.00 | 16.86 |
| 3.16. Pelvis | Pelvis | 5.71 | 1.32 | 2.54 | 1.22 | 1.34 | 0.80 | 3.01 | 1.31 | 3.38 | 1.12 | .26 | 1.95 | .39 | 14.68 |
| 3.17. Skull | Cráneo | 4.00 | 1.34 | 3.68 | 1.22 | 1.68 | 1.08 | 3.82 | 1.07 | 3.24 | 1.31 | .43 | 1.16 | .54 | 15.63 |
| 3.18. Toe | Uña | 1.90 | 0.84 | 4.90 | 0.34 | 3.61 | 1.51 | 3.70 | 1.11 | 2.16 | 1.04 | .95 | 0.30 | .95 | 15.13 |
| 3.19. Tongue | Lengua | 1.75 | 0.71 | 4.80 | 0.61 | 1.33 | 0.80 | 4.25 | 0.94 | 2.46 | 1.28 | .92 | 0.22 | .92 | 18.54 |
| 3.20. Vertebra | Vértebra | 5.41 | 1.33 | 2.74 | 1.43 | 1.38 | 0.84 | 2.81 | 1.34 | 2.44 | 1.25 | .34 | 2.12 | .34 | 13.11 |
| ***4. FLOWERS*** |  |  |  |  |  |  |  |  |  |  |  |  |  |  |  |
| 4.1. Araceae | Cala | 5.63 | 1.80 | 2.86 | 1.41 | 1.84 | 1.04 | 2.84 | 1.28 | 2.11 | 0.92 | .23 | 3.01 | .23 | 16.43 |
| 4.2. Bellflowers | Campanillas | 5.09 | 1.67 | 2.55 | 1.15 | 1.67 | 1.02 | 2.92 | 1.39 | 2.96 | 1.01 | .16 | 3.06 | .35 | 14.67 |
| 4.3. Carnation | Clavel | 3.57 | 1.35 | 3.90 | 1.07 | 2.43 | 1.21 | 4.57 | 0.59 | 2.71 | 1.11 | .85 | 0.77 | .85 | 14.81 |
| 4.4. Daisy | Margarita | 2.52 | 0.93 | 4.42 | 0.76 | 2.24 | 1.28 | 4.84 | 0.44 | 2.06 | 0.94 | .95 | 0.33 | .95 | 17.38 |
| 4.5. Geranium | Geranio | 3.97 | 1.43 | 3.57 | 1.23 | 2.38 | 1.31 | 3.94 | 1.12 | 3.52 | 1.16 | .18 | 2.26 | .38 | 13.51 |
| 4.6. Lilac | Lilas | 4.96 | 1.60 | 2.81 | 1.22 | 2.00 | 1.15 | 3.29 | 1.25 | 3.33 | 1.12 | .26 | 2.46 | .26 | 14.25 |
| 4.7. Orchid | Orquídea | 5.67 | 1.47 | 2.71 | 1.13 | 2.04 | 1.20 | 3.61 | 1.07 | 3.41 | 1.03 | .36 | 1.85 | .36 | 14.34 |
| 4.8. Pansy | Pensamiento | 5.43 | 1.77 | 2.75 | 1.37 | 2.00 | 1.12 | 3.33 | 1.30 | 2.89 | 1.04 | .30 | 1.61 | .42 | 18.17 |
| 4.9. Poppy | Amapola | 3.37 | 1.58 | 4.03 | 0.96 | 1.75 | 0.99 | 4.27 | 0.81 | 2.47 | 0.90 | .89 | 0.56 | .89 | 14.74 |
| 4.10. Rose | Rosa | 2.71 | 1.13 | 4.37 | 0.74 | 2.37 | 1.27 | 4.87 | 0.44 | 2.73 | 1.26 | .98 | 0.17 | .98 | 19.02 |
| 4.11. Sunflower | Girasol | 3.01 | 1.15 | 3.81 | 1.06 | 2.12 | 1.28 | 3.55 | 1.16 | 2.40 | 1.10 | .56 | 1.60 | .56 | 15.77 |
| 4.12. Tulip | Tulipán | 4.28 | 1.39 | 3.42 | 1.20 | 1.92 | 1.15 | 3.87 | 0.92 | 2.22 | 0.95 | .61 | 1.42 | .61 | 13.66 |
| ***5. FRUITS*** |  |  |  |  |  |  |  |  |  |  |  |  |  |  |  |
| 5.1. Apple | Manzana | 1.95 | 0.88 | 4.87 | 0.47 | 3.95 | 1.26 | 4.92 | 0.27 | 1.46 | 0.78 | .99 | 0.00 | .99 | 17.20 |
| 5.2. Avocado | Aguacate | 5.52 | 1.48 | 3.24 | 1.39 | 3.80 | 1.11 | 2.63 | 1.24 | 1.85 | 0.83 | .70 | 0.74 | .70 | 15.32 |
| 5.3. Banana | Plátano | 1.95 | 0.88 | 4.79 | 0.52 | 3.99 | 1.24 | 4.83 | 0.41 | 1.58 | 0.80 | .98 | 0.17 | .98 | 15.65 |
| 5.4. Cherries | Cerezas | 2.97 | 1.18 | 4.42 | 0.85 | 3.38 | 1.42 | 4.27 | 0.83 | 1.73 | 0.86 | .95 | 0.27 | .95 | 15.24 |
| 5.5. Coconut | Coco | 3.44 | 1.29 | 3.22 | 1.21 | 3.56 | 1.28 | 2.75 | 1.16 | 1.86 | 0.93 | .75 | 0.76 | .75 | 16.88 |
| 5.6. Custard apple | Chirimoya | 5.04 | 1.83 | 3.00 | 1.42 | 3.81 | 1.25 | 2.85 | 1.30 | 2.26 | 1.17 | .51 | 1.92 | .51 | 14.13 |
| 5.7. Fig | Higo | 3.44 | 1.29 | 3.72 | 1.13 | 3.53 | 1.29 | 3.10 | 1.16 | 1.70 | 0.92 | .80 | 0.62 | .80 | 14.15 |
| 5.8. Flat peach | Paraguaya | 5.31 | 1.64 | 3.03 | 1.30 | 3.78 | 1.18 | 2.90 | 1.31 | 2.40 | 1.00 | .41 | 2.62 | .41 | 15.32 |
| 5.9. Grapes | Uvas | 2.29 | 1.03 | 4.42 | 0.83 | 3.76 | 1.26 | 4.29 | 0.70 | 2.11 | 0.83 | .96 | 0.27 | .96 | 16.00 |
| 5.10. Kiwi fruit | Kiwi | 5.16 | 1.90 | 4.25 | 1.01 | 3.71 | 1.31 | 3.81 | 1.06 | 1.59 | 0.90 | .96 | 0.11 | .96 | 15.05 |
| 5.11. Lemon | Limón | 2.25 | 0.84 | 4.65 | 0.66 | 3.58 | 1.31 | 4.10 | 1.12 | 1.41 | 0.73 | .90 | 0.52 | .90 | 16.90 |
| 5.12. Mango | Mango | 5.80 | 1.40 | 2.53 | 1.21 | 3.75 | 1.26 | 2.72 | 1.26 | 1.66 | 0.88 | .27 | 2.26 | .27 | 16.74 |
| 5.13. Melon | Melón | 2.34 | 0.85 | 4.65 | 0.68 | 3.99 | 1.09 | 4.49 | 0.73 | 1.57 | 0.73 | 1.00 | 0.00 | 1.00 | 15.40 |
| 5.14. Orange | Naranja | 1.87 | 0.82 | 4.84 | 0.41 | 4.09 | 1.11 | 4.82 | 0.57 | 1.18 | 0.45 | 1.00 | 0.00 | 1.00 | 18.16 |
| 5.15. Peach | Melocotón | 2.54 | 0.87 | 4.29 | 1.00 | 3.52 | 1.39 | 4.53 | 0.66 | 1.47 | 0.75 | .89 | 0.65 | .89 | 14.85 |
| 5.16. Pear | Pera | 2.05 | 0.73 | 4.58 | 0.76 | 3.71 | 1.30 | 4.84 | 0.44 | 1.66 | 0.81 | 1.00 | 0.00 | 1.00 | 16.15 |
| 5.17. Pomegranate | Granada | 4.37 | 1.58 | 3.56 | 1.25 | 3.94 | 1.15 | 3.00 | 1.10 | 1.89 | 0.78 | .84 | 0.22 | .84 | 19.47 |
| 5.18. Quince | Membrillo | 4.24 | 1.64 | 3.11 | 1.29 | 3.58 | 1.25 | 2.70 | 1.17 | 1.63 | 0.74 | .41 | 2.12 | .41 | 14.66 |
| 5.19. Redcurrant | Grosella | 6.06 | 1.36 | 2.18 | 1.07 | 3.26 | 1.43 | 2.14 | 1.08 | 2.99 | 1.09 | .14 | 3.26 | .14 | 13.14 |
| 5.20. Strawberry | Fresa | 2.54 | 1.10 | 4.58 | 0.69 | 3.56 | 1.37 | 4.67 | 0.61 | 2.03 | 1.01 | .94 | 0.31 | .94 | 16.32 |
| 5.21. Watermelon | Sandía | 2.51 | 0.78 | 4.49 | 0.85 | 3.80 | 1.30 | 4.41 | 0.69 | 1.67 | 0.89 | 97 | 0.18 | 97 | 14.82 |
| ***6. INSECTS*** |  |  |  |  |  |  |  |  |  |  |  |  |  |  |  |
| 6.1. Ant | Hormiga | 2.03 | 0.77 | 4.46 | 0.89 | 1.61 | 0.88 | 4.51 | 0.75 | 2.08 | 1.00 | .96 | 0.20 | .96 | 15.45 |
| 6.2. Bee | Abeja | 2.90 | 1.03 | 3.46 | 1.13 | 1.32 | 0.76 | 4.35 | 0.82 | 3.24 | 1.23 | .61 | 1.75 | .61 | 15.55 |
| 6.3. Beetle | Escarabajo | 3.37 | 1.35 | 3.05 | 1.02 | 1.33 | 0.89 | 3.94 | 1.07 | 2.84 | 1.07 | .82 | 0.85 | .82 | 15.43 |
| 6.4. Butterfly | Mariposa | 2.28 | 0.97 | 4.17 | 0.86 | 1.38 | 0.72 | 4.12 | 1.06 | 2.59 | 1.21 | .99 | 0.10 | .99 | 16.55 |
| 6.5. Centipede | Ciempiés | 3.49 | 1.35 | 2.47 | 1.04 | 1.24 | 0.65 | 3.17 | 1.21 | 3.12 | 1.13 | .83 | 0.73 | .83 | 12.79 |
| 6.6. Cockroach | Cucaracha | 3.01 | 1.17 | 3.27 | 1.17 | 1.47 | 0.86 | 4.18 | 0.93 | 3.08 | 1.12 | .48 | 1.80 | .48 | 14.45 |
| 6.7. Dragonfly | Libélula | 4.26 | 1.62 | 2.87 | 1.09 | 1.33 | 0.71 | 3.32 | 1.13 | 3.23 | 1.06 | .46 | 1.87 | .46 | 13.74 |
| 6.8. Fly | Mosca | 2.13 | 0.77 | 4.59 | 0.65 | 1.77 | 1.15 | 4.78 | 0.52 | 3.03 | 1.22 | .99 | 0.10 | .99 | 16.25 |
| 6.9. Grasshopper | Saltamontes | 3.14 | 1.19 | 3.35 | 1.09 | 1.47 | 0.94 | 3.99 | 1.11 | 3.96 | 1.27 | .76 | 0.99 | .76 | 14.27 |
| 6.10. Ladybird | Mariquita | 2.47 | 0.92 | 3.77 | 1.09 | 1.65 | 1.07 | 3.96 | 1.00 | 2.53 | 1.14 | .95 | 0.10 | .95 | 14.31 |
| 6.11. Mosquito | Mosquito | 2.63 | 1.00 | 4.32 | 0.91 | 1.77 | 1.06 | 4.75 | 0.65 | 2.37 | 1.03 | .81 | 0.71 | .81 | 15.32 |
| 6.12. Moth | Polilla | 4.22 | 1.52 | 3.24 | 1.22 | 1.38 | 0.79 | 3.62 | 1.04 | 3.22 | 1.16 | .67 | 1.21 | .67 | 14.19 |
| 6.13. Praying mantis | Mantis | 5.44 | 1.47 | 2.29 | 1.17 | 1.11 | 0.32 | 3.20 | 1.28 | 3.61 | 1.09 | .68 | 1.32 | .68 | 14.19 |
| 6.14. Scorpion | Escorpión | 3.94 | 1.27 | 2.49 | 1.22 | 1.25 | 0.71 | 2.61 | 1.27 | 3.49 | 1.13 | .84 | 0.66 | .84 | 15.19 |
| 6.15. Spider | Araña | 2.38 | 0.87 | 3.57 | 1.28 | 1.33 | 0.69 | 4.24 | 0.94 | 2.46 | 1.08 | .79 | 0.74 | .79 | 16.28 |
| 6.16. Termite | Termita | 5.33 | 1.43 | 1.80 | 0.91 | 1.22 | 0.52 | 2.67 | 1.26 | 2.87 | 1.03 | .27 | 2.27 | .27 | 12.51 |
| 6.17. Wasp | Avispa | 2.87 | 1.05 | 4.01 | 1.09 | 1.45 | 0.91 | 4.49 | 0.68 | 3.13 | 1.23 | .88 | 0.54 | .88 | 14.58 |
| ***7. M.CREATURES*** |  |  |  |  |  |  |  |  |  |  |  |  |  |  |  |
| 7.1. Cockle | Berberecho | 4.67 | 1.62 | 3.34 | 1.08 | 3.61 | 1.25 | 2.63 | 1.19 | 2.18 | 0.90 | .16 | 1.89 | .47 | 12.82 |
| 7.2. Crab | Cangrejo | 3.14 | 1.24 | 3.16 | 1.23 | 2.20 | 1.36 | 3.49 | 1.19 | 3.18 | 1.05 | .93 | 0.22 | .93 | 15.40 |
| 7.3. Dolphin | Delfín | 3.01 | 1.08 | 3.19 | 1.29 | 1.34 | 0.70 | 4.47 | 0.73 | 2.33 | 0.97 | .99 | 0.11 | .99 | 15.64 |
| 7.4. Eel | Anguila | 5.48 | 1.48 | 1.72 | 0.80 | 1.57 | 1.00 | 2.33 | 1.21 | 1.76 | 1.22 | .18 | 3.73 | .18 | 15.82 |
| 7.5. Goose barnacle | Percebe | 5.82 | 1.41 | 2.59 | 1.33 | 3.72 | 1.35 | 2.39 | 1.24 | 3.61 | 0.98 | .67 | 0.62 | .67 | 13.50 |
| 7.6. Killer whale | Orca | 4.41 | 1.62 | 2.74 | 1.24 | 1.22 | 0.66 | 3.72 | 1.15 | 2.59 | 1.04 | .55 | 1.55 | .55 | 14.12 |
| 7.7. Lobster | Bogavante | 5.70 | 1.54 | 2.90 | 1.27 | 3.13 | 1.41 | 2.87 | 1.21 | 3.66 | 1.12 | .19 | 2.20 | .30 | 14.38 |
| 7.8. Manatee | Manatí | 6.51 | 1.07 | 1.64 | 0.90 | 1.23 | 0.64 | 1.96 | 1.18 | 2.88 | 1.02 | .08 | 2.86 | .25 | 13.40 |
| 7.9 .Mussel | Mejillón | 3.77 | 1.14 | 3.99 | 0.98 | 3.57 | 1.28 | 3.06 | 1.26 | 1.80 | 0.84 | .92 | 0.51 | .92 | 13.91 |
| 7.10. Narwhal | Narval | 6.62 | 1.02 | 1.32 | 0.81 | 1.14 | 0.55 | 1.76 | 1.25 | 3.13 | 1.09 | .03 | 3.03 | .15 | 13.40 |
| 7.11. Oyster | Ostra | 5.24 | 1.41 | 2.47 | 1.08 | 3.18 | 1.39 | 2.49 | 1.15 | 3.19 | 1.37 | .38 | 2.54 | .38 | 13.94 |
| 7.12. Pomfret | Palometa | 6.13 | 1.39 | 2.13 | 1.25 | 2.60 | 1.51 | 2.32 | 1.10 | 2.91 | 1.11 | .11 | 2.32 | .49 | 13.87 |
| 7.13. Ray | Raya | 5.06 | 1.61 | 2.44 | 1.13 | 1.67 | 1.17 | 3.19 | 1.24 | 3.38 | 1.10 | .54 | 1.92 | .54 | 16.48 |
| 7.14. Razor-shell | Navaja | 5.47 | 1.57 | 2.84 | 1.23 | 3.72 | 1.17 | 2.15 | 1.09 | 1.89 | 1.06 | .78 | 0.23 | .78 | 15.46 |
| 7.15. Shark | Tiburón | 3.16 | 1.26 | 2.87 | 1.40 | 1.27 | 0.69 | 4.42 | 0.79 | 2.63 | 1.03 | .89 | 0.58 | .89 | 15.69 |
| 7.16. Sperm whale | Cachalote | 5.21 | 1.69 | 2.10 | 1.01 | 1.24 | 0.69 | 3.09 | 1.27 | 2.64 | 1.03 | .24 | 1.50 | .55 | 12.81 |
| 7.17. Starfish | Estrella de mar | 3.52 | 1.29 | 2.66 | 1.19 | 1.23 | 0.62 | 3.58 | 1.05 | 2.49 | 1.23 | 1.00 | 0.00 | 1.00 | 18.58 |
| 7.18. Whale | Ballena | 3.11 | 1.32 | 2.99 | 1.28 | 1.28 | 0.68 | 4.32 | 1.02 | 2.70 | 0.98 | .44 | 1.98 | .44 | 15.73 |
| ***8. NUTS*** |  |  |  |  |  |  |  |  |  |  |  |  |  |  |  |
| 8.1. Acorn | Bellota | 3.82 | 1.65 | 3.10 | 1.23 | 2.77 | 1.35 | 2.63 | 1.30 | 2.38 | 0.94 | .71 | 1.00 | .71 | 15.64 |
| 8.2. Almond | Almendra | 3.10 | 1.10 | 3.84 | 1.13 | 3.65 | 1.29 | 4.42 | 0.90 | 1.81 | 0.82 | .63 | 1.30 | .63 | 15.32 |
| 8.3. Chestnut | Castaña | 2.95 | 1.11 | 3.89 | 0.93 | 3.96 | 1.09 | 3.71 | 1.10 | 1.53 | 0.84 | .72 | 1.09 | .72 | 14.97 |
| 8.4. Date | Dátil | 4.92 | 1.52 | 2.85 | 1.37 | 3.19 | 1.36 | 2.77 | 1.26 | 2.24 | 1.17 | .40 | 1.92 | .40 | 12.59 |
| 8.5. Hazelnut | Avellana | 3.70 | 1.23 | 3.39 | 1.15 | 3.38 | 1.37 | 4.24 | 0.92 | 1.54 | 0.80 | .61 | 1.69 | .61 | 14.23 |
| 8.6. Peanut | Cacahuete | 2.87 | 1.00 | 4.14 | 0.92 | 4.11 | 1.09 | 4.58 | 0.61 | 1.85 | 0.82 | .92 | 0.46 | .92 | 13.73 |
| 8.7. Pine kernel | Piñón | 3.91 | 1.60 | 3.05 | 1.08 | 3.65 | 1.17 | 3.27 | 1.21 | 1.77 | 1.07 | .08 | 2.77 | .24 | 15.09 |
| 8.8. Pipe | Pipa | 2.54 | 0.89 | 4.46 | 1.00 | 4.32 | 1.04 | 4.38 | 0.92 | 1.85 | 0.96 | .82 | 0.55 | .82 | 15.63 |
| 8.9. Pistachio | Pistacho | 4.34 | 1.48 | 4.04 | 0.94 | 4.15 | 1.03 | 4.13 | 0.91 | 2.09 | 0.83 | .76 | 0.81 | .76 | 13.94 |
| 8.10. Raisin | Pasa | 4.09 | 1.43 | 3.64 | 1.09 | 3.29 | 1.25 | 3.71 | 1.22 | 2.46 | 1.14 | .83 | 0.40 | .83 | 19.23 |
| 8.11. Walnut | Nuez | 3.04 | 1.11 | 4.39 | 0.87 | 3.94 | 1.18 | 4.56 | 0.73 | 2.06 | 0.97 | .97 | 0.00 | .97 | 15.81 |
| ***9. TREES*** |  |  |  |  |  |  |  |  |  |  |  |  |  |  |  |
| 9.1. Black poplar | Chopo | 4.48 | 1.65 | 3.24 | 1.22 | 1.61 | 0.82 | 3.73 | 1.09 | 2.22 | 0.87 | .01 | 1.76 | .57 | 13.98 |
| 9.2. Cedar | Cedro | 5.64 | 1.50 | 2.41 | 0.96 | 1.59 | 1.00 | 2.90 | 1.26 | 2.95 | 0.95 | .01 | 1.67 | .45 | 15.35 |
| 9.3. Cypress | Ciprés | 4.68 | 1.57 | 3.41 | 1.05 | 1.49 | 0.72 | 3.79 | 1.06 | 2.13 | 1.02 | .55 | 1.55 | .55 | 14.36 |
| 9.4. Eucalyptus | Eucalipto | 5.08 | 1.70 | 2.62 | 1.25 | 1.58 | 0.92 | 3.19 | 1.24 | 2.99 | 0.99 | .08 | 2.20 | .48 | 14.76 |
| 9.5. Fig tree | Higuera | 4.81 | 1.59 | 2.92 | 1.05 | 2.10 | 1.19 | 3.13 | 1.19 | 3.27 | 1.07 | .01 | 1.94 | .50 | 15.59 |
| 9.6. Fir | Abeto | 4.06 | 1.47 | 3.21 | 1.17 | 1.82 | 1.15 | 3.94 | 1.05 | 3.13 | 0.99 | .39 | 1.64 | .39 | 14.27 |
| 9.7. Holm oak | Encina | 4.78 | 1.62 | 3.13 | 1.25 | 1.68 | 0.90 | 3.85 | 1.06 | 3.12 | 1.13 | .31 | 2.32 | .31 | 15.40 |
| 9.8. Olive tree | Olivo | 4.12 | 1.44 | 3.76 | 1.16 | 2.24 | 1.34 | 4.18 | 0.85 | 3.17 | 1.02 | .53 | 1.67 | .53 | 15.88 |
| 9.9. Palm tree | Palmera | 3.11 | 1.14 | 3.82 | 1.02 | 1.61 | 0.95 | 3.68 | 1.04 | 2.75 | 1.07 | .97 | 0.06 | .97 | 15.67 |
| 9.10. Pine tree | Pino | 2.89 | 1.38 | 4.25 | 0.81 | 1.59 | 0.93 | 4.61 | 0.71 | 2.19 | 0.89 | .58 | 1.37 | .58 | 17.45 |
| 9.11. Willow | Sauce | 4.71 | 1.69 | 3.37 | 1.23 | 1.56 | 0.92 | 3.80 | 1.17 | 3.08 | 1.10 | .66 | 1.10 | .66 | 15.51 |
| ***10. VEGETABLES*** |  |  |  |  |  |  |  |  |  |  |  |  |  |  |  |
| 10.1. Artichoke | Alcachofa | 4.00 | 1.49 | 3.81 | 1.12 | 3.44 | 1.38 | 3.81 | 0.95 | 2.35 | 0.88 | .79 | 0.74 | .79 | 14.76 |
| 10.2. Asparagus | Espárrago | 4.03 | 1.40 | 4.08 | 0.94 | 3.54 | 1.31 | 3.85 | 0.97 | 1.59 | 0.73 | .85 | 0.59 | .85 | 14.12 |
| 10.3. Cabbage | Repollo | 4.49 | 1.64 | 3.63 | 1.20 | 3.78 | 1.24 | 3.77 | 1.15 | 2.10 | 0.89 | .28 | 1.94 | .28 | 14.43 |
| 10.4. Carrot | Zanahoria | 2.54 | 1.02 | 4.47 | 0.88 | 3.72 | 1.18 | 4.33 | 0.78 | 1.28 | 0.53 | 1.00 | 0.00 | 1.00 | 15.87 |
| 10.5. Cauliflower | Coliflor | 3.75 | 1.25 | 4.08 | 0.93 | 3.59 | 1.25 | 4.16 | 0.81 | 2.53 | 0.97 | .85 | 0.67 | .85 | 15.18 |
| 10.6. Celery | Apio | 5.32 | 1.52 | 3.43 | 1.23 | 3.76 | 1.13 | 3.33 | 1.09 | 2.19 | 0.77 | .61 | 1.57 | .61 | 14.99 |
| 10.7. Chard | Acelgas | 4.04 | 1.51 | 3.99 | 1.15 | 3.63 | 1.32 | 4.24 | 0.85 | 2.28 | 0.85 | .72 | 1.16 | .72 | 14.53 |
| 10.8. Cucumber | Pepino | 3.05 | 1.12 | 4.39 | 0.93 | 3.77 | 1.14 | 4.03 | 0.96 | 1.44 | 0.67 | .97 | 0.17 | .97 | 15.76 |
| 10.9. Eggplant | Berenjena | 4.16 | 1.44 | 3.94 | 1.19 | 3.48 | 1.34 | 3.66 | 1.10 | 1.71 | 0.72 | .93 | 0.11 | .93 | 14.87 |
| 10.10. Endive | Escarola | 5.09 | 1.70 | 3.35 | 1.20 | 3.61 | 1.20 | 3.46 | 1.16 | 3.01 | 1.17 | .54 | 1.59 | .54 | 13.57 |
| 10.11. Leek | Puerro | 4.79 | 1.64 | 3.77 | 1.14 | 3.81 | 1.28 | 4.00 | 1.06 | 2.33 | 0.89 | .65 | 1.72 | .65 | 14.48 |
| 10.12. Lettuce | Lechuga | 2.62 | 0.88 | 4.82 | 0.45 | 4.11 | 1.00 | 4.61 | 0.67 | 2.13 | 0.92 | .97 | 0.10 | .97 | 15.77 |
| 10.13. Onion | Cebolla | 2.72 | 1.00 | 4.66 | 0.62 | 4.19 | 1.01 | 4.08 | 1.03 | 1.47 | 0.66 | .99 | 0.00 | .99 | 16.62 |
| 10.14. Pepper | Pimiento | 3.00 | 1.14 | 4.63 | 0.62 | 3.78 | 1.13 | 4.34 | 0.85 | 1.77 | 0.75 | .99 | 0.00 | .99 | 15.74 |
| 10.15. Potato | Patata | 1.97 | 0.75 | 4.90 | 0.30 | 4.16 | 0.97 | 3.78 | 1.17 | 1.28 | 0.58 | 1.00 | 0.00 | 1.00 | 16.03 |
| 10.16. Pumpkin | Calabaza | 3.96 | 1.41 | 3.53 | 1.12 | 3.36 | 1.35 | 3.35 | 1.13 | 1.67 | 0.98 | .83 | 0.57 | .83 | 15.81 |
| 10.17. Red cabbage | Lombarda | 5.91 | 1.39 | 2.68 | 1.41 | 3.42 | 1.28 | 2.64 | 1.23 | 2.32 | 1.49 | .33 | 2.29 | .33 | 13.62 |
| 10.18. Spinach | Espinaca | 3.78 | 1.38 | 3.85 | 1.08 | 3.69 | 1.29 | 4.44 | 0.80 | 2.41 | 1.05 | .46 | 2.09 | .46 | 15.57 |
| 10.19. Tomato | Tomate | 2.09 | 0.66 | 4.81 | 0.51 | 4.05 | 1.02 | 4.51 | 0.86 | 1.23 | 0.45 | 1.00 | 0.00 | 1.00 | 17.15 |
| 10.20. Turnip | Nabo | 5.59 | 1.45 | 1.94 | 1.02 | 3.18 | 1.33 | 2.41 | 1.19 | 2.32 | 1.26 | .08 | 3.09 | .08 | 14.54 |
| 11. BUILDINGS |  |  |  |  |  |  |  |  |  |  |  |  |  |  |  |
| 11.1. Castle | Castillo | 2.62 | 0.92 | 3.54 | 1.14 | 2.11 | 1.52 | 3.05 | 1.21 | 3.19 | 1.24 | .96 | 0.32 | .96 | 18.34 |
| 11.2. Cathedral | Catedral | 4.32 | 1.27 | 3.81 | 1.02 | 3.18 | 1.76 | 3.82 | 0.94 | 4.49 | 0.96 | .81 | 0.62 | .81 | 17.46 |
| 11.3. Church | Iglesia | 2.39 | 0.74 | 4.27 | 0.97 | 2.48 | 1.68 | 3.73 | 1.01 | 3.23 | 1.17 | .85 | 0.71 | .85 | 18.68 |
| 11.4. Factory | Fábrica | 3.86 | 1.33 | 3.41 | 1.04 | 3.52 | 1.65 | 2.97 | 1.18 | 4.43 | 0.97 | .63 | 1.60 | .63 | 17.64 |
| 11.5. Granary | Hórreo | 6.17 | 1.25 | 2.15 | 1.30 | 2.92 | 1.50 | 1.63 | 0.85 | 3.69 | 0.93 | .51 | 1.74 | .51 | 13.75 |
| 11.6. House | Casa | 1.43 | 0.65 | 4.90 | 0.30 | 2.95 | 1.54 | 4.77 | 0.68 | 2.67 | 1.24 | .93 | 0.41 | .93 | 20.31 |
| 11.7. Lighthouse | Faro | 3.97 | 1.24 | 2.95 | 1.01 | 2.08 | 1.41 | 2.14 | 0.92 | 2.77 | 1.11 | .96 | 0.25 | .96 | 17.55 |
| 11.8. Mill | Molino | 3.25 | 0.98 | 3.39 | 1.22 | 2.41 | 1.45 | 1.90 | 0.93 | 2.92 | 1.00 | .97 | 0.20 | .97 | 16.46 |
| 11.9. Pagoda | Pagoda | 6.51 | 1.02 | 1.58 | 0.83 | 1.99 | 1.48 | 1.73 | 1.00 | 4.03 | 0.91 | .26 | 1.76 | .39 | 13.46 |
| 11.10. Palace | Palacio | 3.73 | 1.40 | 2.96 | 1.30 | 3.04 | 1.76 | 3.44 | 1.12 | 4.40 | 1.09 | .33 | 2.94 | .33 | 18.30 |
| 11.11. Pyramid | Pirámide | 3.90 | 1.23 | 3.14 | 1.23 | 2.58 | 1.76 | 1.81 | 0.98 | 2.13 | 1.07 | .84 | 0.40 | .84 | 16.07 |
| 11.12. Shanty | Chabola | 5.05 | 1.52 | 2.71 | 1.31 | 3.53 | 1.49 | 1.97 | 0.99 | 3.47 | 1.08 | .79 | 1.09 | .79 | 13.14 |
| 11.13. Silo | Silo | 6.11 | 1.33 | 1.73 | 1.05 | 2.11 | 1.34 | 1.57 | 0.86 | 2.44 | 1.18 | .29 | 2.59 | .29 | 14.57 |
| 11.14. Skyscraper | Rascacielos | 3.95 | 1.20 | 3.77 | 1.14 | 2.53 | 1.74 | 4.46 | 0.98 | 3.29 | 1.11 | .73 | 0.96 | .73 | 15.75 |
| 11.15. Tower | Torre | 3.03 | 1.15 | 3.43 | 1.07 | 2.48 | 1.61 | 3.28 | 1.07 | 3.15 | 1.05 | .78 | 0.78 | .78 | 18.31 |
| ***12. CLOTHING*** |  |  |  |  |  |  |  |  |  |  |  |  |  |  |  |
| 12.1. Bathrobe | Albornoz | 3.63 | 1.35 | 4.56 | 0.66 | 3.99 | 1.02 | 2.77 | 1.15 | 2.05 | 0.93 | .91 | 0.39 | .91 | 15.28 |
| 12.2. Biretta | Birrete | 6.47 | 1.02 | 2.18 | 1.03 | 2.87 | 1.32 | 1.56 | 0.93 | 2.43 | 0.92 | .36 | 2.32 | .36 | 12.34 |
| 12.3. Cap | Gorra | 2.47 | 0.97 | 4.23 | 0.97 | 3.28 | 1.24 | 3.25 | 1.16 | 1.72 | 0.66 | .96 | 0.25 | .96 | 15.74 |
| 12.4. Clog | Zueco | 4.51 | 1.57 | 2.58 | 1.33 | 3.22 | 1.40 | 1.75 | 0.94 | 2.44 | 0.81 | .75 | 1.07 | .75 | 13.02 |
| 12.5. Coat | Abrigo | 2.18 | 0.93 | 4.58 | 0.71 | 3.59 | 1.18 | 4.71 | 0.53 | 2.14 | 0.84 | .96 | 0.21 | .96 | 16.04 |
| 12.6. Glove | Guante | 2.49 | 0.96 | 4.61 | 0.63 | 4.68 | 0.74 | 3.76 | 0.87 | 2.01 | 0.85 | 1.00 | 0.00 | 1.00 | 15.80 |
| 12.7. Jacket | Chaqueta | 3.06 | 1.11 | 4.47 | 0.81 | 4.06 | 1.00 | 4.72 | 0.53 | 2.10 | 0.87 | .70 | 1.03 | .70 | 16.20 |
| 12.8. Shirt | Camisa | 2.39 | 1.08 | 4.78 | 0.50 | 4.10 | 1.09 | 4.85 | 0.40 | 2.23 | 0.88 | .99 | 0.10 | .99 | 16.48 |
| 12.9. Shoe | Zapato | 1.86 | 1.00 | 4.87 | 0.37 | 4.03 | 1.09 | 4.47 | 0.73 | 1.90 | 0.91 | 1.00 | 0.00 | 1.00 | 15.80 |
| 12.10. Skirt | Falda | 2.28 | 0.90 | 4.54 | 0.73 | 3.79 | 1.23 | 4.60 | 0.65 | 1.26 | 0.61 | .99 | 0.10 | .99 | 16.31 |
| 12.11. Socks | Calcetín | 1.86 | 0.71 | 4.91 | 0.33 | 4.30 | 0.90 | 4.22 | 0.92 | 1.43 | 0.57 | 1.00 | 0.00 | 1.00 | 13.96 |
| 12.12. Trousers | Pantalón | 1.77 | 0.72 | 4.92 | 0.27 | 3.80 | 1.20 | 4.95 | 0.22 | 1.58 | 0.63 | 1.00 | 0.00 | 1.00 | 16.13 |
| 12.13. Undershirt | Camiseta | 2.08 | 1.03 | 4.79 | 0.52 | 3.82 | 1.29 | 4.63 | 0.72 | 1.37 | 0.63 | .99 | 0.00 | .99 | 17.52 |
| ***13. DESK MATERIAL*** |  |  |  |  |  |  |  |  |  |  |  |  |  |  |  |
| 13.1. Compasses | Compás | 3.62 | 0.95 | 4.05 | 0.93 | 4.68 | 0.54 | 3.65 | 0.95 | 2.34 | 0.97 | .97 | 0.00 | .97 | 15.73 |
| 13.2. Eraser | Borrador | 2.23 | 0.75 | 4.57 | 0.80 | 4.77 | 0.51 | 4.62 | 0.56 | 1.22 | 0.50 | .04 | 1.31 | .90 | 16.58 |
| 13.3. Felt-tip pen | Rotulador | 2.78 | 1.23 | 4.58 | 0.63 | 4.77 | 0.60 | 4.32 | 0.76 | 2.01 | 0.85 | .93 | 0.46 | .93 | 13.97 |
| 13.4. Folder | Carpeta | 3.05 | 0.99 | 4.71 | 0.58 | 4.20 | 1.00 | 4.28 | 0.86 | 1.35 | 0.51 | .96 | 0.17 | .96 | 17.12 |
| 13.5. Fountain pen | Pluma | 3.90 | 1.18 | 3.76 | 1.05 | 4.66 | 0.64 | 4.15 | 0.83 | 2.16 | 0.82 | .88 | 0.66 | .88 | 16.65 |
| 13.6. Ink pad | Tampón | 5.71 | 1.34 | 3.37 | 1.39 | 4.18 | 0.99 | 3.17 | 1.24 | 2.18 | 0.99 | .28 | 2.09 | .28 | 13.38 |
| 13.7. Paperclip | Clip | 4.29 | 1.35 | 4.61 | 0.76 | 4.53 | 0.75 | 4.37 | 0.83 | 1.23 | 0.45 | .99 | 0.11 | .99 | 17.05 |
| 13.8. Pen | Bolígrafo | 2.22 | 0.85 | 4.85 | 0.49 | 4.79 | 0.67 | 4.86 | 0.39 | 1.37 | 0.56 | 1.00 | 0.00 | 1.00 | 15.17 |
| 13.9. Pencil | Lápiz | 1.79 | 0.76 | 4.82 | 0.58 | 4.76 | 0.67 | 4.82 | 0.45 | 1.21 | 0.54 | .99 | 0.10 | .99 | 16.15 |
| 13.10. Pencil sharpener | Sacapuntas | 2.39 | 0.81 | 4.71 | 0.64 | 4.62 | 0.82 | 4.43 | 0.80 | 2.13 | 0.85 | .97 | 0.23 | .97 | 13.91 |
| 13.11. Ruler | Regla | 2.52 | 0.71 | 4.58 | 0.81 | 4.43 | 0.90 | 4.32 | 0.95 | 1.53 | 0.60 | 0.99 | 0.00 | 0.99 | 17.47 |
| 13.12. Set square | Cartabón | 4.35 | 1.37 | 3.68 | 1.12 | 4.42 | 0.84 | 3.23 | 1.19 | 1.85 | 0.80 | .54 | 1.24 | .54 | 12.25 |
| 13.13. Square | Escuadra | 4.48 | 1.27 | 3.70 | 1.07 | 4.38 | 0.91 | 3.37 | 1.17 | 1.73 | 0.78 | .64 | 1.15 | .64 | 15.83 |
| 13.14. Rubber stamp | Sello | 4.78 | 1.37 | 3.44 | 1.11 | 4.39 | 1.04 | 3.37 | 1.16 | 2.18 | 0.87 | .58 | 1.40 | .58 | 17.81 |
| 13.15. Stapler | Grapadora | 3.97 | 1.31 | 4.36 | 0.84 | 4.69 | 0.61 | 4.42 | 0.83 | 3.09 | 1.28 | .93 | 0.10 | .93 | 13.24 |
| ***14. FOOD*** |  |  |  |  |  |  |  |  |  |  |  |  |  |  |  |
| 14.1. Anchovy | Anchoas | 4.16 | 1.21 | 3.87 | 1.11 | 3.65 | 1.21 | 3.05 | 1.05 | 1.94 | 0.85 | .92 | 0.30 | .92 | 14.84 |
| 14.2. Black pudding | Morcilla | 3.71 | 1.41 | 3.97 | 1.06 | 4.04 | 1.19 | 3.64 | 1.06 | 1.82 | 1.14 | .56 | 1.76 | .56 | 14.74 |
| 14.3. Caviar | Caviar | 5.67 | 1.28 | 2.19 | 1.19 | 3.14 | 1.37 | 2.14 | 1.37 | 2.81 | 1.24 | .58 | 1.82 | .58 | 15.12 |
| 14.4. Cheese | Queso | 2.32 | 0.88 | 4.78 | 0.47 | 4.22 | 1.03 | 4.25 | 0.76 | 1.46 | 0.64 | 1.00 | 0.00 | 1.00 | 17.23 |
| 14.5. Chorizo | Chorizo | 2.53 | 0.77 | 4.63 | 0.77 | 4.01 | 1.15 | 4.24 | 0.75 | 1.51 | 0.64 | .94 | 0.39 | .94 | 15.59 |
| 14.6. Cookie | Galleta | 1.59 | 0.69 | 4.82 | 0.39 | 4.17 | 1.01 | 4.03 | 0.87 | 1.51 | 0.82 | .93 | 0.53 | .93 | 14.95 |
| 14.7. Crème caramel | Flan | 2.26 | 1.01 | 4.56 | 0.73 | 4.10 | 1.12 | 3.69 | 0.98 | 1.96 | 0.96 | .96 | 0.27 | .96 | 15.52 |
| 14.8. Fritter | Churro | 2.78 | 1.22 | 4.45 | 0.89 | 4.36 | 1.04 | 3.32 | 1.09 | 1.53 | 0.66 | .96 | 0.20 | .96 | 13.87 |
| 14.9. Large fritter | Porra | 3.37 | 1.68 | 4.01 | 1.19 | 4.23 | 1.13 | 2.91 | 1.08 | 1.42 | 0.71 | .58 | 1.05 | .58 | 15.96 |
| 14.10. Millefeuille | Milhojas | 4.63 | 1.52 | 3.29 | 1.25 | 4.27 | 0.94 | 2.47 | 1.19 | 1.94 | 0.81 | .46 | 2.20 | .46 | 11.69 |
| 14.11. NT (Zarajo) | Zarajo | 6.60 | 0.86 | 1.71 | 1.13 | 3.50 | 1.40 | 1.58 | 1.05 | 3.88 | 0.98 | .21 | 2.15 | .21 | 9.01 |
| 14.12. Paella | Paella | 2.96 | 1.07 | 4.54 | 0.83 | 4.19 | 1.11 | 4.63 | 0.74 | 3.58 | 1.52 | 1.00 | 0.00 | 1.00 | 16.21 |
| 14.13. Pasty | Empanadilla | 3.11 | 1.12 | 4.20 | 0.94 | 4.22 | 1.07 | 3.72 | 1.09 | 2.04 | 0.87 | .90 | 0.42 | .90 | 12.69 |
| 14.14. Pie | Empanada | 3.94 | 1.42 | 4.00 | 1.06 | 4.37 | 0.94 | 3.66 | 0.92 | 2.03 | 0.93 | .73 | 1.43 | .73 | 15.29 |
| 14.15. Steak | Filete | 2.64 | 1.27 | 4.65 | 0.68 | 4.05 | 1.19 | 4.72 | 0.58 | 3.06 | 1.25 | .31 | 2.45 | .31 | 15.35 |
| 15. FURNITURE |  |  |  |  |  |  |  |  |  |  |  |  |  |  |  |
| 15.1. Armchair | Sillón | 2.43 | 0.94 | 4.70 | 0.69 | 2.44 | 1.36 | 4.56 | 0.78 | 2.23 | 0.82 | .71 | 1.12 | .71 | 16.15 |
| 15.2. Bed | Cama | 1.47 | 0.60 | 4.89 | 0.53 | 3.37 | 1.33 | 4.68 | 0.65 | 2.32 | 0.88 | 1.00 | 0.00 | 1.00 | 18.06 |
| 15.3. Bedside table | Mesilla | 3.17 | 1.28 | 4.23 | 0.95 | 3.40 | 1.31 | 4.12 | 0.82 | 2.68 | 0.99 | .76 | 1.14 | .76 | 13.99 |
| 15.4. Bookcase | Librería | 3.51 | 1.31 | 4.47 | 0.77 | 3.58 | 1.27 | 4.06 | 0.93 | 3.27 | 1.20 | .64 | 1.57 | .64 | 17.77 |
| 15.5. Chair | Silla | 1.74 | 0.81 | 4.78 | 0.62 | 3.18 | 1.36 | 4.83 | 0.47 | 2.08 | 0.95 | 1.00 | 0.00 | 1.00 | 17.41 |
| 15.6. Chest of drawers | Cómoda | 4.46 | 1.65 | 3.96 | 0.95 | 3.67 | 1.31 | 3.81 | 0.90 | 2.81 | 0.98 | .53 | 2.18 | .53 | 16.93 |
| 15.7. Couch | Diván | 5.94 | 1.27 | 2.76 | 1.12 | 2.35 | 1.38 | 2.71 | 1.14 | 2.91 | 1.07 | .59 | 1.72 | .59 | 14.70 |
| 15.8. Filling cabinet | Archivador | 4.91 | 1.42 | 3.90 | 1.06 | 4.32 | 0.82 | 2.84 | 1.09 | 2.52 | 0.92 | .76 | 1.31 | .76 | 13.62 |
| 15.9. Lamp | Lámpara | 2.43 | 0.81 | 4.61 | 0.63 | 3.47 | 1.21 | 3.59 | 1.04 | 2.52 | 0.96 | 1.00 | 0.00 | 1.00 | 16.20 |
| 15.10. Lectern | Atril | 6.04 | 1.30 | 2.94 | 1.34 | 3.18 | 1.19 | 2.05 | 1.04 | 2.73 | 1.10 | .29 | 2.46 | .29 | 14.63 |
| 15.11. Rocking chair | Mecedora | 3.85 | 1.41 | 3.54 | 1.10 | 3.09 | 1.30 | 3.28 | 1.07 | 3.06 | 1.23 | .86 | 0.66 | .86 | 13.65 |
| 15.12. Sofa | Sofá | 2.47 | 1.19 | 4.71 | 0.68 | 2.28 | 1.36 | 4.65 | 0.73 | 2.43 | 1.03 | .81 | 0.89 | .81 | 16.45 |
| 15.13. Stool | Taburete | 3.20 | 1.23 | 4.35 | 0.80 | 2.97 | 1.30 | 3.56 | 0.97 | 2.08 | 0.96 | .96 | 0.32 | .96 | 14.33 |
| 15.14. Table | Mesa | 1.73 | 0.71 | 4.91 | 0.40 | 3.03 | 1.40 | 4.90 | 0.35 | 1.53 | 0.62 | 1.00 | 0.00 | 1.00 | 18.97 |
| 15.15. Wardrobe | Armario | 2.23 | 0.85 | 4.78 | 0.50 | 3.63 | 1.10 | 4.81 | 0.39 | 1.68 | 0.78 | 1.00 | 0.00 | 1.00 | 16.81 |
| ***16. JEWELLERY*** |  |  |  |  |  |  |  |  |  |  |  |  |  |  |  |
| 16.1. Bangle/Bracelet | Esclava | 4.96 | 1.75 | 3.23 | 1.22 | 3.95 | 1.21 | 3.43 | 1.18 | 2.71 | 0.96 | .57 | 1.27 | .57 | 15.09 |
| 16.2. Bracelet | Pulsera | 3.03 | 1.04 | 3.86 | 1.14 | 3.91 | 1.17 | 4.58 | 0.78 | 2.85 | 1.01 | .92 | 0.60 | .92 | 16.07 |
| 16.3. Brooch | Broche | 4.85 | 1.54 | 2.97 | 1.22 | 3.90 | 1.17 | 3.67 | 1.12 | 4.03 | 1.14 | .47 | 2.32 | .47 | 15.71 |
| 16.4. Cufflinks | Gemelos | 5.47 | 1.53 | 3.05 | 1.27 | 4.37 | 0.84 | 2.77 | 1.10 | 3.00 | 1.10 | .70 | 0.92 | .70 | 16.35 |
| 16.5. Diadem | Diadema | 3.67 | 1.43 | 2.85 | 1.21 | 3.63 | 1.17 | 3.01 | 1.19 | 2.91 | 1.13 | .34 | 1.48 | .48 | 14.38 |
| 16.6. Diamond | Diamante | 4.51 | 1.44 | 2.68 | 1.26 | 3.19 | 1.38 | 4.67 | 0.83 | 3.10 | 1.24 | .78 | 1.10 | .78 | 16.58 |
| 16.7. Medal | Medalla | 3.16 | 1.20 | 3.66 | 1.29 | 2.94 | 1.36 | 3.71 | 1.24 | 2.95 | 1.26 | .71 | 0.90 | .71 | 17.13 |
| 16.8. Necklace | Collar | 2.63 | 0.99 | 4.22 | 0.98 | 3.77 | 1.21 | 4.62 | 0.72 | 2.09 | 0.87 | .99 | 0.10 | .99 | 16.25 |
| 16.9. Pendant | Pendiente | 2.75 | 1.06 | 4.30 | 0.91 | 4.24 | 0.85 | 4.61 | 0.74 | 2.22 | 0.90 | .95 | 0.20 | .95 | 17.75 |
| 16.10. Ring | Anillo | 2.92 | 1.12 | 4.20 | 1.11 | 4.34 | 1.12 | 4.73 | 0.55 | 2.61 | 1.02 | .82 | 0.66 | .82 | 17.13 |
| 16.11. Seal ring | Sello | 4.65 | 1.75 | 3.13 | 1.36 | 4.16 | 1.13 | 3.51 | 1.07 | 2.37 | 0.94 | .42 | 1.14 | .55 | 17.81 |
| 16.12. Tie clip | Pisa corbatas | 5.76 | 1.74 | 2.64 | 1.39 | 4.17 | 1.04 | 1.97 | 0.95 | 2.64 | 1.13 | .31 | 2.36 | .31 | 11.50 |
| ***17. KITCHEN UT.*** |  |  |  |  |  |  |  |  |  |  |  |  |  |  |  |
| 17.1. Cooking pot | Puchero | 4.15 | 1.42 | 2.91 | 1.29 | 3.76 | 1.15 | 3.08 | 1.17 | 1.91 | 0.74 | .14 | 3.59 | .15 | 14.53 |
| 17.2. Cup | Taza | 1.96 | 0.82 | 4.70 | 0.70 | 4.46 | 0.93 | 4.13 | 1.05 | 1.59 | 0.71 | .82 | 0.90 | .82 | 16.43 |
| 17.3. Fondue | Fondue | 6.42 | 1.08 | 2.71 | 1.15 | 4.54 | 0.66 | 2.16 | 1.09 | 3.77 | 1.05 | .74 | 0.70 | .74 | 14.19 |
| 17.4. Fork | Tenedor | 1.79 | 0.83 | 4.97 | 0.16 | 4.88 | 0.53 | 4.85 | 0.46 | 1.83 | 0.90 | 1.00 | 0.00 | 1.00 | 15.21 |
| 17.5. Frying pan | Sartén | 3.05 | 1.16 | 4.78 | 0.53 | 4.54 | 0.72 | 4.85 | 0.40 | 1.67 | 0.70 | .99 | 0.10 | .99 | 15.65 |
| 17.6. NT (Churrera) | Churrera | 5.82 | 1.38 | 1.73 | 1.03 | 4.43 | 0.92 | 1.67 | 0.96 | 3.15 | 0.93 | .11 | 2.83 | .11 | 11.82 |
| 17.7. Peeler | Pelador | 5.47 | 1.53 | 3.33 | 1.23 | 4.80 | 0.61 | 3.30 | 1.16 | 2.33 | 0.81 | .49 | 1.90 | .49 | 12.57 |
| 17.8. Pot | Olla | 3.27 | 1.35 | 4.50 | 0.75 | 4.14 | 1.09 | 4.59 | 0.59 | 1.83 | 0.75 | .69 | 1.32 | .69 | 16.36 |
| 17.9. Saucepan | Cacerola | 3.05 | 1.19 | 4.62 | 0.65 | 4.10 | 1.07 | 4.68 | 0.52 | 1.92 | 0.78 | .46 | 1.60 | .46 | 14.92 |
| 17.10. Small saucepan | Cazo | 3.01 | 1.14 | 4.61 | 0.77 | 4.43 | 0.89 | 4.53 | 0.71 | 1.82 | 0.67 | .68 | 1.29 | .68 | 14.90 |
| 17.11. Sharpening steel | Afilador | 4.87 | 1.48 | 3.20 | 1.19 | 4.72 | 0.58 | 2.71 | 1.10 | 1.81 | 0.70 | .39 | 2.45 | .39 | 13.58 |
| 17.12. Strainer | Colador | 3.47 | 1.25 | 4.35 | 0.85 | 4.37 | 0.86 | 4.19 | 0.64 | 2.43 | 0.94 | .89 | 0.40 | .89 | 14.21 |
| 17.13. Teapot | Tetera | 4.15 | 1.42 | 3.58 | 1.14 | 4.20 | 0.91 | 3.20 | 1.15 | 2.63 | 0.88 | .86 | 0.58 | .86 | 14.76 |
| ***18. MUSICAL INST.*** |  |  |  |  |  |  |  |  |  |  |  |  |  |  |  |
| 18.1. Accordion | Acordeón | 4.03 | 1.45 | 2.87 | 1.14 | 4.57 | 1.01 | 3.63 | 1.11 | 3.62 | 1.15 | .97 | 0.00 | .97 | 15.04 |
| 18.2. Balalaika | Balalaica | 6.73 | 0.76 | 1.52 | 0.95 | 4.80 | 0.49 | 1.94 | 1.29 | 4.29 | 0.98 | .13 | 2.82 | .16 | 9.93 |
| 18.3. Bugle | Corneta | 4.72 | 1.76 | 2.71 | 1.09 | 4.47 | 0.85 | 3.04 | 1.19 | 2.32 | 0.83 | .30 | 1.20 | .61 | 14.17 |
| 18.4. Clarinet | Clarinete | 5.27 | 1.39 | 2.65 | 1.27 | 4.76 | 0.56 | 3.87 | 1.00 | 3.25 | 1.13 | .53 | 1.47 | .53 | 15.05 |
| 18.5. Drum | Tambor | 2.58 | 0.94 | 3.30 | 1.19 | 4.58 | 0.73 | 4.28 | 0.85 | 2.84 | 1.11 | .92 | 0.49 | .92 | 15.96 |
| 18.6. Flute | Flauta | 3.10 | 1.06 | 3.97 | 1.13 | 4.81 | 0.43 | 4.59 | 0.63 | 1.59 | 0.67 | 1.00 | 0.00 | 1.00 | 16.06 |
| 18.7. Guitar | Guitarra | 2.76 | 0.91 | 4.27 | 0.98 | 4.84 | 0.37 | 4.89 | 0.32 | 2.76 | 0.98 | 1.00 | 0.00 | 1.00 | 17.74 |
| 18.8. Harmonica | Armónica | 3.84 | 1.22 | 3.32 | 1.15 | 4.54 | 0.89 | 3.37 | 1.11 | 2.54 | 1.01 | .92 | 0.00 | .92 | 15.55 |
| 18.9. Harp | Arpa | 4.30 | 1.37 | 2.73 | 1.22 | 4.82 | 0.38 | 3.58 | 1.01 | 3.33 | 1.12 | .92 | 0.30 | .92 | 15.19 |
| 18.10. Maracas | Maracas | 4.59 | 1.36 | 2.70 | 1.11 | 4.59 | 0.91 | 3.05 | 1.15 | 2.38 | 1.08 | .96 | 0.11 | .96 | 13.85 |
| 18.11. Piano | Piano | 3.16 | 1.22 | 3.51 | 1.21 | 4.73 | 0.76 | 4.66 | 0.68 | 3.81 | 1.37 | .96 | 0.25 | .96 | 17.32 |
| 18.12. Saxophone | Saxofón | 4.80 | 1.33 | 2.99 | 1.26 | 4.75 | 0.67 | 4.14 | 0.93 | 4.16 | 1.09 | .87 | 0.54 | .87 | 14.77 |
| 18.13. Tambourine | Pandereta | 2.10 | 0.78 | 4.15 | 1.06 | 4.81 | 0.43 | 3.39 | 0.99 | 2.70 | 1.30 | .99 | 0.00 | .99 | 14.28 |
| 18.14. Trumpet | Trompeta | 3.32 | 1.24 | 3.29 | 1.08 | 4.49 | 1.08 | 4.54 | 0.64 | 3.37 | 1.29 | .97 | 0.21 | .97 | 15.81 |
| 18.15. Tuba | Tuba | 5.82 | 1.36 | 2.24 | 1.19 | 4.73 | 0.59 | 3.19 | 1.24 | 3.73 | 1.11 | .14 | 2.24 | .37 | 14.26 |
| 18.16. Violin | Violín | 3.74 | 1.52 | 3.31 | 1.20 | 4.82 | 0.58 | 4.60 | 0.61 | 3.47 | 1.36 | .86 | 0.66 | .86 | 15.96 |
| ***19. SPORTS/GAMES*** |  |  |  |  |  |  |  |  |  |  |  |  |  |  |  |
| 19.1. Soccer ball | Balón | 1.79 | 0.80 | 4.63 | 0.67 | 3.29 | 1.37 | 4.79 | 0.49 | 1.65 | 0.92 | .85 | 0.58 | .85 | 17.45 |
| 19.2. Ball | Pelota | 1.45 | 0.70 | 4.60 | 0.80 | 4.24 | 0.79 | 4.64 | 0.66 | 2.32 | 1.38 | .89 | 0.56 | .89 | 17.47 |
| 19.3. Chess | Ajedrez | 3.67 | 1.09 | 4.03 | 0.97 | 4.57 | 0.81 | 3.72 | 1.05 | 3.49 | 1.34 | 1.00 | 0.00 | 1.00 | 17.13 |
| 19.4. Dart | Dardo | 4.28 | 1.39 | 3.28 | 1.06 | 4.68 | 0.59 | 2.92 | 0.92 | 2.38 | 0.87 | .95 | 0.20 | .95 | 15.26 |
| 19.5. Dartboard | Diana | 3.74 | 1.35 | 3.64 | 1.07 | 3.94 | 1.28 | 3.29 | 0.94 | 3.21 | 1.40 | .95 | 0.20 | .95 | 17.37 |
| 19.6. Diabolo | Diábolo | 4.91 | 1.68 | 2.81 | 1.30 | 4.88 | 0.39 | 2.50 | 1.13 | 2.71 | 1.02 | .59 | 0.85 | .59 | 13.36 |
| 19.7. Dice | Dado | 2.65 | 0.91 | 4.36 | 0.88 | 4.67 | 0.66 | 3.73 | 1.09 | 1.97 | 1.09 | 1.00 | 0.00 | 1.00 | 19.18 |
| 19.8. Doll | Muñeca | 1.59 | 0.61 | 4.20 | 1.13 | 4.25 | 0.90 | 3.80 | 1.43 | 3.22 | 1.22 | 1.00 | 0.00 | 1.00 | 16.45 |
| 19.9. Jump rope | Comba | 2.68 | 0.92 | 3.71 | 1.13 | 4.64 | 0.76 | 3.55 | 1.11 | 2.41 | 1.21 | .65 | 1.27 | .65 | 14.89 |
| 19.10. Ludo | Parchís | 2.65 | 0.88 | 4.41 | 0.88 | 4.56 | 0.86 | 3.90 | 1.20 | 3.81 | 1.28 | .99 | 0.00 | .99 | 14.23 |
| 19.11. Racket | Raqueta | 3.12 | 1.27 | 4.15 | 0.94 | 4.82 | 0.45 | 4.50 | 0.72 | 2.10 | 0.96 | 1.00 | 0.00 | 1.00 | 15.40 |
| 19.12. Skate | Patín | 2.91 | 0.91 | 3.78 | 1.09 | 3.58 | 1.12 | 3.56 | 0.89 | 3.46 | 1.26 | .97 | 0.20 | .97 | 15.39 |
| 19.13. Ski | Esquís | 4.22 | 1.44 | 3.04 | 1.31 | 3.41 | 1.28 | 3.71 | 0.96 | 2.27 | 0.97 | .81 | 0.41 | .81 | 14.06 |
| 19.14. Skittle | Bolo | 3.83 | 1.27 | 3.40 | 1.00 | 3.38 | 1.48 | 3.10 | 1.04 | 1.67 | 0.85 | .95 | 0.00 | .95 | 15.18 |
| 19.15. Spinning top | Peonza | 2.91 | 1.05 | 3.73 | 1.09 | 4.59 | 0.81 | 2.94 | 1.17 | 1.81 | 0.70 | .84 | 0.72 | .84 | 13.38 |
| 19.16. Table football | Futbolín | 3.53 | 1.10 | 4.03 | 0.93 | 4.72 | 0.78 | 3.37 | 1.19 | 3.66 | 1.39 | .93 | 0.36 | .93 | 14.59 |
| ***20. TOOLS*** |  |  |  |  |  |  |  |  |  |  |  |  |  |  |  |
| 20.1. Axe | Hacha | 3.18 | 1.11 | 3.67 | 1.11 | 4.70 | 0.70 | 3.82 | 1.00 | 1.85 | 0.68 | .96 | 0.20 | .96 | 15.53 |
| 20.2. Bit | Broca | 5.15 | 1.64 | 3.58 | 1.23 | 4.28 | 0.89 | 3.61 | 1.08 | 1.77 | 0.75 | .73 | 0.97 | .73 | 13.89 |
| 20.3. Chisel | Formón | 6.10 | 1.25 | 2.16 | 1.23 | 4.51 | 1.01 | 2.75 | 1.42 | 2.29 | 0.94 | .15 | 3.13 | .19 | 11.17 |
| 20.4. Cold chisel | Cortafríos | 6.08 | 1.40 | 2.18 | 1.25 | 4.39 | 1.14 | 2.52 | 1.24 | 1.53 | 0.93 | .13 | 2.69 | .25 | 11.33 |
| 20.5. Hammer | Martillo | 2.76 | 0.84 | 4.40 | 0.73 | 4.79 | 0.49 | 4.85 | 0.49 | 1.77 | 0.84 | 1.00 | 0.00 | 1.00 | 15.84 |
| 20.6. Handsaw | Serrucho | 3.83 | 1.45 | 3.42 | 1.16 | 4.78 | 0.50 | 4.33 | 0.75 | 1.81 | 0.81 | .65 | 0.93 | .65 | 13.41 |
| 20.7. Leveller | Nivel | 5.54 | 1.58 | 3.16 | 1.39 | 4.38 | 0.77 | 3.27 | 1.26 | 2.85 | 1.00 | .68 | 0.83 | .68 | 19.79 |
| 20.8. Nail | Clavo | 3.05 | 1.07 | 4.06 | 1.09 | 4.35 | 0.88 | 4.00 | 1.13 | 1.19 | 0.51 | .76 | 0.85 | .76 | 16.00 |
| 20.9. Nut | Tuerca | 3.91 | 1.33 | 3.75 | 1.14 | 4.24 | 1.00 | 3.77 | 1.10 | 2.04 | 0.90 | .81 | 0.98 | .81 | 15.60 |
| 20.10. Pincers | Alicates | 4.11 | 1.53 | 4.01 | 0.91 | 4.63 | 0.74 | 4.51 | 0.77 | 2.41 | 0.82 | .89 | 0.32 | .89 | 14.19 |
| 20.11. Pliers | Tenazas | 4.32 | 1.25 | 3.37 | 1.11 | 4.70 | 0.70 | 3.94 | 1.00 | 1.89 | 0.80 | .63 | 1.10 | .63 | 13.67 |
| 20.12. Screw | Tornillo | 3.34 | 1.12 | 4.13 | 0.97 | 4.37 | 0.82 | 3.92 | 1.21 | 1.96 | 0.85 | .90 | 0.37 | .90 | 15.42 |
| 20.13. Screwdriver | Destornillador | 3.37 | 1.17 | 4.43 | 0.73 | 4.62 | 0.77 | 4.81 | 0.48 | 1.65 | 0.72 | .94 | 0.25 | .94 | 14.27 |
| 20.14. Shovel | Pala | 2.78 | 0.94 | 3.59 | 1.21 | 4.63 | 0.75 | 4.23 | 0.96 | 1.86 | 0.78 | .96 | 0.00 | .96 | 16.01 |
| 20.15. Trowel | Llana | 5.76 | 1.47 | 2.46 | 1.43 | 4.61 | 0.90 | 3.04 | 1.38 | 2.20 | 0.95 | .32 | 2.50 | .32 | 15.02 |
| ***21. VEHICLES*** |  |  |  |  |  |  |  |  |  |  |  |  |  |  |  |
| 21.1. Boat | Barca | 2.63 | 1.05 | 3.37 | 1.24 | 3.76 | 1.25 | 2.42 | 1.08 | 2.25 | 0.88 | .86 | 0.78 | .86 | 18.33 |
| 21.2. Bus | Autobús | 2.42 | 0.83 | 4.52 | 0.80 | 4.04 | 1.42 | 4.58 | 0.65 | 2.62 | 1.00 | .76 | 0.78 | .76 | 17.29 |
| 21.3. Car | Coche | 1.90 | 1.04 | 4.86 | 0.42 | 4.68 | 0.73 | 4.94 | 0.37 | 3.17 | 1.34 | .93 | 0.53 | .93 | 19.12 |
| 21.4. Cart | Carro | 2.85 | 1.11 | 3.05 | 1.24 | 3.27 | 1.31 | 2.37 | 1.19 | 3.38 | 1.19 | .75 | 1.10 | .75 | 17.49 |
| 21.5. Motorbike | Moto | 2.49 | 1.29 | 4.05 | 1.04 | 4.60 | 0.71 | 4.63 | 0.54 | 3.67 | 1.41 | .99 | 0.10 | .99 | 18.32 |
| 21.6. Paragliding | Parapente | 5.74 | 1.49 | 2.60 | 1.11 | 4.32 | 1.00 | 1.54 | 0.78 | 3.40 | 1.17 | .43 | 1.41 | .43 | 15.43 |
| 21.7. Plane | Avión | 2.19 | 0.82 | 4.33 | 0.92 | 3.66 | 1.55 | 3.86 | 1.06 | 2.87 | 1.17 | 1.00 | 0.00 | 1.00 | 17.94 |
| 21.8. Scooter | Patinete | 3.15 | 1.24 | 3.49 | 1.23 | 4.32 | 0.99 | 2.04 | 0.91 | 2.38 | 0.87 | .81 | 0.87 | .81 | 13.61 |
| 21.9. Ship | Barco | 2.44 | 1.10 | 3.54 | 1.17 | 3.95 | 1.25 | 3.35 | 1.19 | 3.50 | 1.31 | .74 | 1.33 | .74 | 17.94 |
| 21.10. Skateboard | Monopatín | 3.81 | 1.55 | 3.56 | 1.04 | 3.12 | 1.33 | 1.81 | 0.95 | 1.79 | 0.80 | .73 | 1.09 | .73 | 13.41 |
| 21.11. Tractor | Tractor | 3.05 | 1.23 | 3.34 | 1.21 | 4.19 | 1.12 | 3.13 | 1.33 | 3.80 | 1.39 | .99 | 0.00 | .99 | 15.71 |
| 21.12. Train | Tren | 2.34 | 1.18 | 4.51 | 0.68 | 2.94 | 1.60 | 4.28 | 0.92 | 3.44 | 1.39 | .93 | 0.41 | .93 | 18.17 |
| 21.13. Van | Furgoneta | 3.14 | 1.35 | 3.97 | 1.10 | 4.35 | 0.98 | 4.26 | 0.80 | 2.54 | 1.10 | .95 | 0.33 | .95 | 16.11 |
| ***22. WEAPONS*** |  |  |  |  |  |  |  |  |  |  |  |  |  |  |  |
| 22.1. Armour | Armadura | 4.11 | 1.23 | 2.43 | 1.26 | 3.27 | 1.48 | 2.56 | 1.29 | 3.77 | 1.17 | .88 | 0.23 | .88 | 15.41 |
| 22.2. Arrow | Flecha | 2.84 | 0.98 | 2.86 | 1.32 | 4.13 | 1.04 | 3.30 | 1.23 | 1.28 | 0.55 | .94 | 0.35 | .94 | 16.40 |
| 22.3. Bayonet | Bayoneta | 6.01 | 1.18 | 1.54 | 0.86 | 3.97 | 1.04 | 2.30 | 1.14 | 2.29 | 0.98 | .20 | 1.25 | .20 | 13.54 |
| 22.4. Boomerang | Bumerán | 4.75 | 1.51 | 2.32 | 1.16 | 4.48 | 1.10 | 1.46 | 0.69 | 1.53 | 0.81 | .77 | 0.35 | .77 | 12.44 |
| 22.5. Bow | Arco | 3.13 | 0.90 | 2.88 | 1.23 | 4.65 | 0.75 | 3.00 | 1.22 | 1.55 | 0.88 | .99 | 0.00 | .99 | 17.67 |
| 22.6. Cannon | Cañón | 3.53 | 1.21 | 2.76 | 1.29 | 4.18 | 0.99 | 3.96 | 1.01 | 2.95 | 1.16 | .98 | 0.10 | .98 | 16.34 |
| 22.7. Crossbow | Ballesta | 5.38 | 1.49 | 1.96 | 1.05 | 4.46 | 1.10 | 2.69 | 1.20 | 3.00 | 1.09 | .53 | 1.53 | .53 | 14.99 |
| 22.8. Grenade | Granada | 4.92 | 1.44 | 2.14 | 1.30 | 4.10 | 1.26 | 3.63 | 1.10 | 2.95 | 1.02 | .89 | 0.58 | .89 | 19.47 |
| 22.9. Gun | Pistola | 2.80 | 0.98 | 3.28 | 1.48 | 4.65 | 0.79 | 4.85 | 0.62 | 2.78 | 1.07 | .90 | 0.40 | .90 | 16.54 |
| 22.10. Helmet | Casco | 3.42 | 1.37 | 2.68 | 1.25 | 3.19 | 1.29 | 2.04 | 1.13 | 2.12 | 0.88 | .79 | 0.70 | .79 | 17.62 |
| 22.11. Machine gun | Ametralladora | 4.57 | 1.50 | 2.23 | 1.32 | 4.42 | 1.08 | 4.34 | 0.99 | 3.56 | 1.15 | .51 | 1.92 | .51 | 14.19 |
| 22.12. Revolver | Revólver | 3.58 | 1.46 | 3.05 | 1.33 | 4.72 | 0.58 | 4.60 | 0.69 | 2.92 | 1.11 | .39 | 0.96 | .61 | 14.81 |
| 22.13. Shield | Escudo | 3.72 | 1.15 | 2.47 | 1.15 | 3.47 | 1.39 | 2.97 | 1.20 | 3.25 | 1.31 | .96 | 0.32 | .96 | 17.12 |
| 22.14. Slingshot | Tirachinas | 3.15 | 0.94 | 3.24 | 1.27 | 4.79 | 0.61 | 2.08 | 1.08 | 2.59 | 1.11 | .95 | 0.30 | .95 | 13.53 |
| 22.15. Sword | Espada | 2.70 | 1.04 | 3.06 | 1.29 | 4.56 | 0.94 | 4.04 | 1.08 | 1.80 | 0.76 | .97 | 0.21 | .97 | 16.94 |
| ***23. NATURE*** |  |  |  |  |  |  |  |  |  |  |  |  |  |  |  |
| 23.1. Cliff | Acantilado | 4.58 | 1.42 | 3.13 | 1.13 | 1.03 | 0.16 | 3.85 | 1.13 | 3.81 | 1.28 | .69 | 1.61 | .69 | 15.27 |
| 23.2. Cloud | Nube | 1.84 | 0.78 | 4.78 | 0.52 | 1.01 | 0.11 | 4.20 | 1.02 | 1.99 | 0.99 | 1.00 | 0.00 | 1.00 | 18.18 |
| 23.3. Coal | Carbón | 3.30 | 1.33 | 2.48 | 1.23 | 2.61 | 1.39 | 2.58 | 1.26 | 2.14 | 1.16 | .29 | 2.27 | .29 | 16.69 |
| 23.4. Gold | Oro | 3.78 | 1.58 | 2.80 | 1.31 | 2.90 | 1.52 | 2.03 | 1.09 | 3.78 | 1.11 | .29 | 2.72 | .29 | 18.99 |
| 23.5. Ice | Hielo | 2.95 | 1.26 | 3.20 | 1.32 | 1.62 | 0.98 | 3.35 | 1.13 | 3.27 | 1.21 | .11 | 1.76 | .11 | 17.47 |
| 23.6. Iceberg | Iceberg | 4.69 | 1.36 | 2.83 | 1.34 | 1.26 | 0.71 | 3.27 | 1.20 | 2.81 | 1.22 | .81 | 0.87 | .81 | 15.14 |
| 23.7. Island | Isla | 2.92 | 0.95 | 3.58 | 1.24 | 1.23 | 0.62 | 3.62 | 1.12 | 2.71 | 1.16 | .84 | 0.74 | .84 | 18.68 |
| 23.8. Moon | Luna | 1.90 | 0.89 | 4.74 | 0.55 | 1.05 | 0.27 | 3.78 | 1.28 | 1.69 | 1.01 | .95 | 0.17 | .95 | 18.73 |
| 23.9. Mountain | Montaña | 2.51 | 1.15 | 4.15 | 0.97 | 1.37 | 0.90 | 4.60 | 0.59 | 2.68 | 1.35 | .83 | 1.01 | .83 | 18.24 |
| 23.10. Puddle | Charco | 2.22 | 1.05 | 4.05 | 1.25 | 1.15 | 0.48 | 2.70 | 1.45 | 2.92 | 1.36 | .26 | 2.74 | .26 | 16.69 |
| 23.11. Sea | Mar | 1.99 | 0.96 | 4.44 | 0.78 | 1.58 | 1.05 | 4.56 | 0.92 | 1.63 | 1.06 | .93 | 0.45 | .93 | 19.93 |
| 23.12. Stone | Piedra | 1.94 | 1.19 | 4.38 | 1.00 | 2.34 | 1.38 | 3.75 | 1.19 | 2.25 | 0.99 | .44 | 2.08 | .44 | 18.10 |
| 23.13. Sun | Sol | 1.52 | 0.80 | 4.70 | 0.91 | 1.13 | 0.46 | 4.54 | 0.92 | 2.18 | 1.28 | .75 | 1.30 | .75 | 19.24 |
| 23.14. Volcano | Volcán | 3.35 | 1.09 | 2.94 | 1.36 | 1.09 | 0.43 | 3.77 | 1.10 | 2.32 | 0.99 | .99 | 0.00 | .99 | 16.33 |
| 23.15. Waterfall | Catarata | 3.96 | 1.24 | 3.04 | 1.18 | 1.14 | 0.61 | 4.11 | 0.99 | 3.92 | 1.32 | .67 | 1.07 | .67 | 14.90 |
| 23.16. Wave | Ola | 2.16 | 1.01 | 4.24 | 0.89 | 1.44 | 0.98 | 3.87 | 0.98 | 2.91 | 1.23 | .89 | 0.70 | .89 | 17.55 |

**Note**: AoA = Age of acquisition; Fam = Familiarity, Man = Manipulability; VC =Visual complexity; NA = Name agreement; LF (natural logarithm) = Lexical frequency. NT = No translation into English.

** Increasing *H* values indicate decreasing name agreement. When *H* = 0 there is a total name agreement, that means that only a name was given for that stimuli.
