# Supplementary material for: An Ecological Alternative to Snodgrass & Vanderwart: 360 High Quality Colour Images with Norms for Seven Psycholinguistic Variables
Source: PLoS One. 2012 May 25;7(5):e37527. doi: 10.1371/journal.pone.0037527 (PMC3360784; doi:10.1371/journal.pone.0037527)
Supplement: Appendix S3 — Proportion (in brackets) of target names, alternative names, acceptable synonyms of each item, plus “Don't know” (DK), “Don't remember” (DR), and “Tip of the tongue” (TOT) responses. (DOC) [file pone.0037527.s003.doc]

| **Item** | **Target name (Spanish)** | **Alternative/synonyms name-s (Spanish)** |
| --- | --- | --- |
|  |  |  |
| ***1. ANIMALS*** |  |  |
| 1.1. Armadillo | Armadillo (57) | Ornitorrinco (4), animal (1), chinchilla (1), melón (1), rata (1), ratón (1), topo (1), zarigüeya (1),  DK (15), DR (9) TOT (8). |
| 1.2. Bat | Murciélago (97) | Lechuza (1).  DR (1) TOT (1). |
| 1.3. Cat | Gato (100) |  |
| 1.4. Cheetah | Guepardo (33) | Leopardo (25), jaguar (10), lince (8), puma (6), pantera (3), felino (1), gacela (1), hiena (1), ibis (1), tigre (1).  TOT (7), DR (3). |
| 1.5. Cow | Vaca (100) |  |
| 1.6. Crocodile | Cocodrilo (78) | Caimán** (19), lagarto (1), reptil (1).  TOT (1). |
| 1.7. Dromedary | Dromedario (50) | Camello (50). |
| 1.8. Elephant | Elefante (100) |  |
| 1.9. Giraffe | Jirafa (98) | Cebra (1).  TOT (1). |
| 1.10. Hippopotamus | Hipopótamo (83) | Rinoceronte (10), cerdo (3), jabalí (1)  DK (1), DR (1), TOT (1). |
| 1.11. Horse | Caballo (99) | Potro** (1). |
| 1.12. Kangaroo | Canguro (100) |  |
| 1.13. Lioness | Leona (59) | León (34), tigre (5), leopardo (2). |
| 1.14. Lynx | Lince (76) | Gato montés (6), felino (1), gato (1), gato salvaje (1), guepardo (1), puma (1), tigre (1).  DK (8), DR (3) TOT (1). |
| 1.15. Platypus | Ornitorrinco (47) | Mamífero (3), morsa (3), topo (3), animal (1), mofeta (1), oso hormiguero (1).  DK (21), DR (13), TOT (7). |
| 1.16. Rhino | Rinoceronte (97) | Hipopótamo (1).  TOT (2). |
| 1.17. Snake | Serpiente (93) | Culebra** (4), bicha** (1).  DR (1), TOT (1). |
| 1.18. Tapir | Tapir (20) | Oso hormiguero (29), okapi (5), ornitorrinco (5), animal (1), cerdo (1), mamífero (1), marsupial (1), oso (1), osobuco (1), rinoceronte (1).  DK (20), DR (10), TOT (4). |
| 1.19. Tiger | Tigre (99) | León (1). |
| 1.20. Turtle | Tortuga (90) | Galápago (10). |
| 1.21. Zebra | Cebra (100) |  |
| ***2. BIRDS*** |  |  |
| 2.1. Barn owl | Lechuza (48) | Búho (46), mochuelo (3), ave (1), halcón (1).  DK (1). |
| 2.2. Duck | Pato (93) | Ánade** (3), oca (2), paloma (1).  TOT (1). |
| 2.3. Goldfinch | Jilguero (34) | Pájaro (28), canario (5), colibrí (4), ave (3), gorrión (3), periquito (3), carbonero (1), petirrojo (1), pinzón (1).  DK (9), DR (7), TOT (1). |
| 2.4. Goose | Oca (52) | Pato (29), ganso** (14), cisne (4).  DR (1). |
| 2.5. Hen | Gallina (87) | Gallo (13). |
| 2.6. Hummingbird | Colibrí (71) | Pájaro (11), ave (3), jilguero (1), ruiseñor (1).  DK (9), TOT (4). |
| 2.7. Kiwi | Kiwi (32) | Pájaro (8), polluelo (8), ave (4), pollo (4), avestruz (3), avutarda (1), buitre (1), grulla (1), ibis (1), pollito (1).  DK (29), DR (6), TOT (1). |
| 2.8. Magpie | Urraca (23) | Pájaro (34), colibrí (5), mirlo (4), gorrión (3), ave (1), cuervo (1), golondrina (1), jilguero (1), martín pescador (1).  DK (11), DR (10), TOT (5). |
| 2.9. Ostrich | Avestruz (97) | Ave (1), pavo (1).  DR (1). |
| 2.10. Owl | Búho (81) | Lechuza (19). |
| 2.11. Partridge | Perdiz (51) | Paloma (16), ave (8), codorniz (4), pájaro (3), faisán (1), pichón (1).  DK (6), DR (6), TOT (4). |
| 2.12. Pelican | Pelícano (70) | Gaviota (3), papagayo (3), ave (1), flamenco (1), ganso (1), pato (1), pavo (1), tucán (1).  DK (8), DR (5), TOT (5). |
| 2.13. Penguin | Pingüino (100) |  |
| 2.14. Pheasant | Faisán (26) | Pavo real (15), pájaro (8), pavo (6), perdiz (6), codorniz (4), urogallo (3), ave (1), pinta (1).  DK (18), DR (6), TOT (6). |
| 2.15. Pigeon | Paloma (100) |  |
| 2.16. Raven | Cuervo (77) | Urraca (7), mirlo (3), pájaro (3), grajo (1), tordo (1).  DK (4), DR (3), TOT (1). |
| 2.17. Rooster | Gallo (90) | Gallina (10). |
| 2.18. Seagull | Gaviota (84) | Paloma (7), pájaro (3).  TOT (4), DK (1), DR (1). |
| 2.19. Sparrow | Gorrión (50) | Pájaro (22), jilguero (11), petirrojo (3), ruiseñor (3), ave (1), golondrina (1).  DK (4), DR (3), TOT (1). |
| 2.20. Toucan | Tucán (58) | Loro (8), cacatúa (6), guacamayo (5), pájaro (6), papagayo (3), pelícano (1), cuervo (1).  DR (5), DK (4), TOT (3). |
| ***3. BODY PARTS*** |  |  |
| 3.1. Arm | Brazo (96) | Antebrazo** (3).  TOT (1). |
| 3.2. Beard | Barba (44) | Piel (3), cuello (1), cuello de visón (1), montera (1).  DK (46), DR (3), TOT (1). |
| 3.3. Bone | Hueso (100) |  |
| 3.4. Brain | Cerebro (86) | Encéfalo (11), sesos** (2).  DK (1). |
| 3.5. Ear | Oreja (99) | Oído (1). |
| 3.6. Eye | Ojo (100) |  |
| 3.7. Finger | Dedo (90) | Índice** (8), dedo índice** (2). |
| 3.8. Foot | Pie (100) |  |
| 3.9. Hand | Mano (100) |  |
| 3.10. Kidney | Riñón (47) | Judía (20), alubia (5), haba (4), hígado (3), pulmón (3), habichuela (1), piedra (1), zapato (1).  DK (14), DR (1). |
| 3.11. Leg | Pierna (100) |  |
| 3.12. Liver | Hígado (69) | Corazón (3), pulmones (3), caramelo (2), carne (1), órgano (1), páncreas (1), riñón (1), roca (1), sesos (1).  DK (11), DR (3), TOT (3). |
| 3.13. Lung | Pulmón (43) | Hígado (19), páncreas (6), víscera (6), riñón (3), bazo (1), corazón (1), lengua (1) órgano (1), pimiento (1).  DK (14), TOT (3), DR (1). |
| 3.14. Mouth | Boca (24) | Labios** (76). |
| 3.15. Nose | Nariz (100) |  |
| 3.16. Pelvis | Pelvis (26) | Cadera (39), cóccix (14), hueso (10), esqueleto (3).  DK (4), TOT (3), DR (1). |
| 3.17. Skull | Cráneo (43) | Calavera** (54), esqueleto (1), huesos cabeza (1).  TOT (1). |
| 3.18. Toe | Uña (95) | Burbuja (2), dedo (1), lengua (1).  DR (1). |
| 3.19. Tongue | Lengua (92) | Col (2), fruto (1).  DK (4), DR (1). |
| 3.20. Vertebra | Vértebra (34) | Hueso (13), pan (5), mortero (4), taba (3), almirez (1), amasador (1), molde (1), yunque (1).  DK (29), TOT (5), DR (3). |
| ***4. FLOWERS*** |  |  |
| 4.1. Araceae | Cala (23) | Flor (15), lirio (15), tulipán (6), azucena (6), gladiolo (3), narciso (3), pétalo (3), campanilla (1), jara (1), manto (1), nardo (1), oreja de burro (1), orquídea (1).  DK (9), TOT (6), DR (5). |
| 4.2. Bellflowers | Campanillas (14) | Flores (35), lilas (8), violetas (8), lirios (6), violeta (5), amapola (1), azaleas (1), azucena (1), dalias (1), galán de noche (1), orquídea (1).  DR (10), DK (4), TOT (4). |
| 4.3. Carnation | Clavel (85) | Rosa (7), flor (3), amapola (1), capullo (1).  TOT (3). |
| 4.4. Daisy | Margarita (95) | Flor (4), amapola (1). |
| 4.5. Geranium | Geranio (20) | Flor (38), rosa (15), clavel (3), petunia (3), begonia (1), gardenia (1), petunias (1), ramo (1), tulipán (1).  DK (8), DR (7), TOT (1). |
| 4.6. Lilac | Lilas (28) | Flores (24), violetas (7), jazmín (4), jacinto (3), glicina (1), hortensia (1), lirio (1), orquídea (1), racimo (1), ramo (1).  DK (14), DR (8), TOT (6). |
| 4.7. Orchid | Orquídea (38) | Flor (28), lila (3), amapola (1), azucena (1), pensamiento (1), petunia (1), tulipán (1), violeta (1).  DR (11), DK (8), TOT (6). |
| 4.8. Pansy | Pensamiento (32) | Flor (42), orquídea (5), petunia (3), geranio (1), violeta (1).  DK (11), DR (4), TOT (1). |
| 4.9. Poppy | Amapola (90) | Flor (5), tulipán (3).  DR (1), TOT (1). |
| 4.10. Rose | Rosa (98) | Capullo (2). |
| 4.11. Sunflower | Girasol (57) | Margarita (25), flor (14), dalia (1), pipero (1), tulipán (1).  DK (1). |
| 4.12. Tulip | Tulipán (61) | Amapola (23), rosa (6), flor (4), clavel (1).  DR (4), TOT (1). |
| ***5. FRUITS*** |  |  |
| 5.1. Apple | Manzana (100) |  |
| 5.2. Avocado | Aguacate (72) | Fruta (4), guacamole (1), kiwi (1), mango (1), papaya (1).  DK (10), DR (5), TOT (5). |
| 5.3. Banana | Plátano (98) | Banana** (2). |
| 5.4. Cherries | Cerezas (95) | Picotas** (3), ciruela (1).  TOT (1). |
| 5.5. Coconut | Coco (77) | Nuez (4), almendra (1), fósil (1), judía (1), semilla (1).  DK (11), DR (3), TOT (1). |
| 5.6. Custard apple | Chirimoya (51) | Aguacate (8), alcachofa (5), fruta (4), verdura (3), breva (1), cogollo (1), granada (1), higo chumbo (1), mango (1), papaya (1).  DK (10), TOT (8), DR (5). |
| 5.7. Fig | Higo (81) | Fruta (3), breva (1), calabacín (1), cebolla (1), membrillo (1)  DK (11), DR (1). |
| 5.8. Flat peach | Paraguaya (42) | Melocotón (15), nectarina (5), albaricoque (4), fresquilla (4), manzana (3), pomelo (3), caqui (1), chirimoya (1), damasco (1), fruta (1), fruto (1), guayaba (1), papaya (1), pera (1).  DR (6), TOT (6), DK (4). |
| 5.9. Grapes | Uvas (96) | Racimo (3), hueso (1). |
| 5.10. Kiwifruit | Kiwi (97) | Melón (1).  DK (1), DR (1). |
| 5.11. Lemon | Limón (90) | Naranja ( 8), membrillo (2). |
| 5.12. Mango | Mango (27) | Fruta (5), papaya (4), aguacate (3), níspero (3), alimento (1), ciruela (1), haba (1), kiwi (1), pomelo (1).  DK (37), DR (11), TOT (5). |
| 5.13. Melon | Melón (100) |  |
| 5.14. Orange | Naranja (100) |  |
| 5.15. Peach | Melocotón (90) | Manzana (4), fruta (3), níspero (1), pomelo (1).  DR (1). |
| 5.16. Pear | Pera (100) |  |
| 5.17. Pomegranate | Granada (84) | Fruta (1), pomelo (1).  DK (9), TOT (4), DR (1). |
| 5.18. Quince | Membrillo (41) | Manzana (22), pomelo (13), mango (5), limón (4), fruta (1), guayaba (1), pera (1).  DK (7), TOT (4), DR (1). |
| 5.19. Redcurrant | Grosella (14) | Arándanos (13), bayas (9), frutos (6), cerezas (5), tomates (4), endrinas (3), uvas (3), ciruela (1), endrina (1), fresas (1), fruta (1), racimo (1), tomate cherry (1).  DR (18), DK (11), TOT (8). |
| 5.20. Strawberry | Fresa (94) | Fresón** (6). |
| 5.21. Watermelon | Sandía (97) | Melón (3). |
| ***6. INSECTS*** |  |  |
| 6.1. Ant | Hormiga (97) | Araña (1), insecto (1).  DR (1). |
| 6.2. Bee | Abeja (62) | Mosca (13), avispa (11), abejorro (7), insecto (3), escarabajo (1), moscardón (1),  DK, (1) TOT (1). |
| 6.3. Beetle | Escarabajo (86) | Cucaracha (10), bicho (1), cascarudo** (1), gorgojo (1), insecto (1).  DR (5). |
| 6.4. Butterfly | Mariposa (99) | Insecto (1). |
| 6.5. Centipede | Ciempiés (83) | Gusano (4), escolopendra** (3), insecto (1), lombriz (1), tijereta (1).  DK (4), DR (3). |
| 6.6. Cockroach | Cucaracha (48) | Escarabajo (19), grillo (14), bicho (4), insecto (3), chinche (1).  DK (9), DR (1), TOT (1). |
| 6.7. Dragonfly | Libélula (46) | Mariposa (27), polilla (8), insecto (4), luciérnaga (3), mosquito (3), aguacil (1).  DK (3), DR (4), TOT (1). |
| 6.8. Fly | Mosca (99) | Mosquito (1). |
| 6.9. Grasshopper | Saltamontes (76) | Cigarra (5), grillo (4), langosta (4), bicho (1), insecto (1).  DR (5), DK (4). |
| 6.10. Ladybird | Mariquita (95) | Mariposa (1).  TOT (3), DR (1). |
| 6.11. Mosquito | Mosquito (82) | Insecto (6), libélula (6).  DK (5), DR (1), |
| 6.12. Moth | Polilla (67) | Mariposa (14), insecto (7), bicho (1), palometa (1).  DR (4), DK (3), TOT (3). |
| 6.13. Praying mantis | Mantis (69) | Saltamontes (14), cigarra (4), insecto (4), picatel (1), santateresa** (1).  DK (6), TOT (1). |
| 6.14. Scorpion | Escorpión (84) | Alacrán** (5), cangrejo (3), escarabajo (3).  TOT (3), DK (1), DR (1). |
| 6.15. Spider | Araña (79) | Tarántula** (21). |
| 6.16. Termite | Termita (27) | Insecto (15), hormiga (14), larva (9), escarabajo (1), gusano (1), hormiga voladora (1), liendre (1).  DK (23), DR (8). |
| 6.17. Wasp | Avispa (88) | Abeja (12). |
| ***7. M. CREATURES*** |  |  |
| 7.1. Cockle | Berberecho (17) | Almeja (47), concha (25), chirla (8), coquina (1), vieira (1).  TOT (1). |
| 7.2. Crab | Cangrejo (96) | Andarica (1), nécora (1).  DK (1), TOT (1). |
| 7.3. Dolphin | Delfín (99) | Tiburón (1). |
| 7.4. Eel | Anguila (20) | Lombriz (13), Judía (6), morena (6), sanguijuela (6), babosa (4), gusano (4), guisante (3), haba (3), judía verde (3), algarroba (1), alubia (1), angula (1), espárrago (1), habichuela (1), lamprea (1), pez (1), serpiente (1), vaina (1), vasija (1).  DK (15), TOT (6), DR (1). |
| 7.5. Goose barnacle | Percebe (69) | Nécora (1), pez (1), pezuña (1), planta (1), vieira (1).  DK (10), TOT (10), DR (6). |
| 7.6. Killer whale | Orca (56) | Ballena (25), delfín (11), tiburón (3), cachalote (1).  TOT (4). |
| 7.7. Lobster | Bogavante (21) | Cangrejo (30), langosta (29), cigala (5), abrecanto** (1), centollo (1), crustáceo (1), escorpión (1), percebe (1).  TOT (9), DR (1). |
| 7.8. Manatee | Manatí (8) | Morsa (25), león marino (18), elefante (9), foca (9), beluga (3), anfibio (1), ballena (1), cetáceo (1), mamífero (1), nutria (1), ocelote (1), oso marino (1).  DR (8), TOT (8), DK (6). |
| 7.9. Mussel | Mejillón (92) | Almeja (3), concha (3), ostra (2). |
| 7.10. Narwhal | Narval (4) | Pez espada (15), ballena (11), delfín (4), foca (4), pez (4), león marino (3), manatí (3), morsa (3), beluga (1), cachalote (1).  DK (29), DR (11), TOT (7). |
| 7.11. Oyster | Ostra (38) | Concha (6), lapa (4), bollo (3), caracol (3), caracola (3), fósil (3), pasa (3), almeja (1), baba (1), caparazón (1), hongo (1), seta (1), torrija (1).  DK (28), DR (3). |
| 7.12. Pomfret | Palometa (10) | Pez (49), pescado (6), besugo (5), dorada (5), atún (3), piraña (3), gallo (1), japuta (1), lenguado (1), pez espada (1), rodaballo (1).  DK (8), DR (5), TOT (1). |
| 7.13. Ray | Raya (54) | Manta (14), pez manta (9), medusa (3), rodaballo (3), babosa (1), caracol (1), carpa (1), mero (1), pez torpedo (1).  DR (5), DK (3), TOT (4). |
| 7.14. Razor-shell | Navaja (78) | Muergo** (2), objeto (1).  DK (19). |
| 7.15. Shark | Tiburón (91) | Pez (4), esturión (1), pez espada (1), trucha (1).  DK (1), DR (1). |
| 7.16. Sperm whale | Cachalote (24) | Ballena (55), tiburón (8), morsa (1), pez (1), pez martillo (1).  DK (6), DR (3), TOT (1). |
| 7.17. Starfish | Estrella de mar (100) |  |
| 7.18. Whale | Ballena (44) | Pez (14), delfín (9), cachalote (5), tiburón (4), orca (3), anguila (1).  DK (12), DR (5), TOT (3). |
| ***8. NUTS*** |  |  |
| 8.1. Acorn | Bellota (71) | Avellana (7), almendra (1), baya (1), dátil (1), nuez (1), semilla (1).  TOT (8), DK (4), DR (3). |
| 8.2. Almond | Almendra (64) | Nuez (11), avellana (8), almendruco (3), fruto (1).  DK (7), DR (4), TOT (2). |
| 8.3. Chestnut | Castaña (72) | Higo (6), avellana (4), breva (3), cebolla (3), remolacha (1).  DK (6), DR (5). |
| 8.4. Date | Dátil (42) | Pasa (24), berenjena (3), arándano (1), cayena (1), ciruela (1), ciruelas (1), crisálida (1), flor (1), ñora (1).  DK (21), TOT (3). |
| 8.5. Hazelnut | Avellana (62) | Castaña (7), calabaza (6), nuez (6), cebolla (3), almendra (1), bellota (1), caqui (1), manzana (1).  DR (6), TOT (4), DR (2). |
| 8.6. Peanut | Cacahuete (93) | Avellana (4), almendra (1), maní** (1).  TOT (1). |
| 8.7. Pine kernel | Piñón (9) | Patata (24), almendra (5), piedra (5), pipa (3), avellana (1), fruto (1), fruto seco (1), guijarro (1), hueso (1), mango (1), papaya (1), pera (1).  DK (42), DR (4). |
| 8.8. Pipe | Pipa (83) | Fruto seco (3), aguacate (1), berenjena (1), papaya (1).  DK (11). |
| 8.9. Pistachio | Pistacho (77) | Almeja (4), bivalvo (1), cacahuete (1), higo (1), nuez (1), panchito (1).  DK (10), TOT (4). |
| 8.10. Raisin | Pasa (83) | Dátil (3), jamón (1), orejón (1).  DK (9), DR (3). |
| 8.11. Walnut | Nuez (100) |  |
| ***9. TREES*** |  |  |
| 9.1. Black poplar | Chopo (1) | Ciprés (60), árbol (14), pino (10), abeto (5), cedro (1), conífera (1), olmo (1).  TOT (4), DK (2), DR (1). |
| 9.2. Cedar | Cedro (1) | Pino (46), abeto (35), árbol (8), chopo (1), pinsapo (1), secuoya (1).  DK (5), DR (1), TOT (1). |
| 9.3. Cypress | Ciprés (55) | Pino (29), árbol (5), abeto (4), arbusto (1), eucalipto (1).  DR (4), TOT (1). |
| 9.4. Eucalyptus | Eucalipto (10) | Árbol (48), álamo (4), abedul (2), chopo (2), ciprés (2), sauce (2), acacia (1), alcornoque (1), bol (1), encina (1), olmo (1), pino (1).  DK (10), DR (10), TOT (4). |
| 9.5. Fig tree | Higuera (1) | Árbol (49), naranjo (6), encina (3), magnolio (3), manzano (3), olivo (3), almendro (1), bol (1), cerezo (1), ciruelo (1), fruta (1).  DR (14), DK (13). |
| 9.6. Fir | Abeto (39) | Pino (37), árbol (15), ciprés (2), abedul (1).  DK (5), DR (1). |
| 9.7. Holm oak | Encina (32) | Árbol (20), olivo (18), roble (3), alcornoque (1), avellano (1), cedro (1), chopo (1), ciprés (1), nogal (1), pino (1).  DK (8), TOT (8), DR (4). |
| 9.8. Olive tree | Olivo (55) | Árbol (23), encina (11), carrasca (1), ciprés (1), nogal (1), roble (1).  DK (4), DR (2), TOT (1). |
| 9.9. Palm tree | Palmera (97) | Árbol (2), palma (1). |
| 9.10. Pine tree | Pino (58) | Árbol (28), alcornoque (2), ciprés (2), humo (1), olmo (1), roble (1).  DK (5), DR (1), TOT (1). |
| 9.11. Willow | Sauce (68) | Árbol (18), ciprés (3), mimosa (1), olmo (1).  DK (5), DR (3), TOT (1). |
| ***10. VEGETABLES*** |  |  |
| 10.1. Artichoke | Alcachofa (79) | Alcaucil** (3), col (3), col de Bruselas (1), coliflor (1), hortaliza (1).  TOT (6), DK (3), DR (3). |
| 10.2. Asparagus | Espárrago (87) | Puerro (3), junco (1), planta (1), rábano (1), verdura (1).  TOT (3), DK (2), DR (1). |
| 10.3. Cabbage | Repollo (30) | Lechuga (35), col (24), coliflor (4), berza (1), iceberg (1).  DR (3), DK (1), TOT (1). |
| 10.4. Carrot | Zanahoria (100) |  |
| 10.5. Cauliflower | Coliflor (86) | Col** (8), repollo (4).  DR (1), TOT (1). |
| 10.6. Celery | Apio (61) | Puerro (13), perejil (8), acelgas (5), cominos (1), nabo (1), verdura (1).  DR (5), DK (4), TOT (1). |
| 10.7. Chard | Acelgas (72) | Lechuga (8), berza (3), espinaca (3), borraja (2), tallos (2), verdura (1).  DK (4), DR (4), TOT (1). |
| 10.8. Cucumber | Pepino (97) | Calabacín (3). |
| 10.9. Eggplant | Berenjena (95) | Calabacín (1).  TOT (3), DR (1). |
| 10.10. Endive | Escarola (56) | Lechuga (21), col (7), berza (1), brócoli (1), ensalada (1), rúcula (1).  DK (4), DR (4), TOT (4). |
| 10.11. Leek | Puerro (65) | Apio (7), cebolla (5), ajo (3), cebolleta (3), cebollino (3), ajete (1), ajo tierno (1), apio (1), esparrago (1), verdura (1).  DR (4), TOT (4), DK (1). |
| 10.12. Lettuce | Lechuga (98) | Berza (1).  TOT (1). |
| 10.13. Onion | Cebolla (100) |  |
| 10.14. Pepper | Pimiento (100) |  |
| 10.15. Potato | Patata (100) |  |
| 10.16. Pumpkin | Calabaza (83) | Calabacín (6), patata (1), rábano (1).  DK (4), DR (4), TOT (1). |
| 10.17. Red cabbage | Lombarda (33) | Col (11), remolacha (5), repollo (3), verdura (3), bola (1), coco (1), col morada (1), escarola (1), higo (1), sandia (1).  DK (22), DR (13), TOT (4). |
| 10.18. Spinach | Espinaca (47) | Acelgas (23), lechuga (10), col (3), berros (1), berza (1), coliflor (1), ensalada (1), hojas (1), perejil (1), planta (1).  DR (6), DK (3), TOT (1). |
| 10.19. Tomato | Tomate (100) |  |
| 10.20. Turnip | Nabo (11) | Fruta (3), patata (3), aceituna (1), batata (1), calabaza (1), lichi (1), limón (1), mango (1), pera (1), tubérculo (1).  DK (64), DR (8) TOT (3). |
| ***11. BUILDINGS*** |  |  |
| 11.1. Castle | Castillo (96) | Fortaleza (2), muralla (1), torreón (1). |
| 11.2. Cathedral | Catedral (83) | Iglesia (11), abadía (2).  DR (2), DK (1), TOT (1). |
| 11.3. Church | Iglesia (85) | Campanario (10), ermita (4).  TOT (1). |
| 11.4. Factory | Fabrica (63) | Chimenea (13), central (4), industria (3), factoría** (3), horno (3), cementerio (1), empresa (1).  DK (6), DR (3). |
| 11.5. Granary | Hórreo (54) | Casa (5), edificio (1), horno (1), lagar (1), mausoleo (1), pajar (1), palacio (1), panteón (1), pazo (1), secador (1), sepultura (1), tumba (1).  DK (19), TOT (6), DR (5). |
| 11.6. House | Casa (93) | Chalet (6), caserío (1). |
| 11.7. Lighthouse | Faro (96) | Torre (4). |
| 11.8. Mill | Molino (97) | Aspas (2), molino de viento** (1). |
| 11.9. Pagoda | Pagoda (27) | Templo (39), edificio (7), casa china (1), casa japonesa (1), estufa (1), torre (1).  DK (10), DR (7), TOT (6). |
| 11.10. Palace | Palacio (33) | Catedral (19), edificio (6), ayuntamiento (5), castillo (5), monasterio (5), iglesia (4), mansión (4), universidad (3), abadía (2), casa (1), facultad (1), monumento (1), palacete (1).  DK (4), DR (4), TOT (2). |
| 11.11. Pyramid | Pirámide (84) | Montaña (3), cemento (1), duna (1).  DK (8), TOT (2), DR (1). |
| 11.12. Shanty | Chabola (79) | Casa (4), choza (4), cabaña (3), caseta (3), casucha (3), chamizo (1).  DK (3). |
| 11.13. Silo | Silo (29) | Torre (13), deposito (7), lámpara (4), faro (3), contenedor (2), edificio (2), molino (2), monumento (2), prisma (2), tanque (2), torreón (1).  DK (29), DR (1), TOT (1). |
| 11.14. Skyscraper | Rascacielos (73) | Edificio (24), torre (3). |
| 11.15. Tower | Torre (79) | Torreón** (10), edificio (3), monumento (1).  DK (5), DR (1), TOT (1). |
| ***12. CLOTHING*** |  |  |
| 12.1. Bathrobe | Albornoz (91) | Bata (8).  TOT (1). |
| 12.2. Biretta | Birrete (37) | Sombrero (14), gorro (9), gorra (4), orla (4), borla (1), capirote (1), toca (1), tocado (1), toga (1).  TOT (11), DR (10), DK (7). |
| 12.3. Cap | Gorra (96) | Visera (4). |
| 12.4. Clog | Zueco (75) | Madreña** (4), zapato (4), albarca** (4), zanco (2), espardeña (1), zapatilla (1).  DR (5), DK (3), TOT (1). |
| 12.5. Coat | Abrigo (97) | Chaquetón (1), gabardina (1).  DR (1). |
| 12.6. Glove | Guante (100) |  |
| 12.7. Jacket | Chaqueta (70) | Americana** (24), traje (4).  TOT (2). |
| 12.8. Shirt | Camisa (99) | Camiseta (1). |
| 12.9. Shoe | Zapato (100) |  |
| 12.10. Skirt | Falda (99) | Mandil (1). |
| 12.11. Socks | Calcetín (100) |  |
| 12.12. Trousers | Pantalón (100) |  |
| 12.13. Undershirt | Camiseta (100) |  |
| ***13. DESK MAT.*** |  |  |
| 13.1. Compasses | Compás (100) |  |
| 13.2. Eraser | Borrador (5) | Goma de borrar** (62), goma** (29), jabón (1), piedra (1).  DK (2). |
| 13.3. Felt-tip pen | Rotulador (94) | Marcador (2), bolígrafo (1), edding (1), permanente (1).  DK (1). |
| 13.4. Folder | Carpeta (96) | Archivador (3).  DK (1). |
| 13.5. Fountain pen | Pluma (88) | Estilográfica** (8), pluma estilográfica** (4). |
| 13.6. Ink pad | Tampón (28) | Tinta (20), tintero (13), sello (3), almohadilla** (1), esponjilla (1), puntero (1), sellador (1).  DR (18), DK (10), TOT (4). |
| 13.7. Paperclip | Clip (99) | Imperdible (1). |
| 13.8. Pen | Bolígrafo (100) |  |
| 13.9. Pencil | Lápiz (99) | Lapicero** (1). |
| 13.10. Pencil sharpener | Sacapuntas (96) | Afilalápices** (4). |
| 13.11. Ruler | Regla (100) |  |
| 13.12. Set square | Cartabón (54) | Escuadra (39), regla (5).  DK (1), TOT (1). |
| 13.13. Square | Escuadra (65) | Cartabón (28), regla (5).  DR (1), TOT (1). |
| 13.14. Rubber stamp | Sello (58) | Tampón (22), peón (3), sellador (3), estampa (1), matasellos (1).  DR (10), TOT (2). |
| 13.15. Stapler | Grapadora (93) | Puntero (1).  DR (4), DK (1), TOT (1). |
| ***14. FOOD*** |  |  |
| 14.1. Anchovy | Anchoas (93) | Boquerón (1), pescado (1), sardinas (1).  TOT (3), DK (1). |
| 14.2. Black pudding | Morcilla (57) | Chorizo (9), salchicha (5), butifarra (3), batata (1), berenjena (1), boniato (1), habichuela (1), judía (1), patata (1), tamarindo (1).  DK (15), DR (3), TOT (1). |
| 14.3. Caviar | Caviar (60) | Moras (10), aceituna (6), arándano (3), ciruelas (1), grosella (1), huevas (1), mancha (1), olivas (1), perlas (1), uvas (1).  DK (13), TOT (1). |
| 14.4. Cheese | Queso (100) |  |
| 14.5. Chorizo | Chorizo (95) | Crisálida (1), guindilla (1), larva (1), morcilla (1).  DK (1). |
| 14.7. Cookie | Galleta (93) | Empanada (3), pan (2), fósil (1), tarta (1). |
| 14.8. Crème caramel | Flan (96) | Postre (3), budín (1). |
| 14.9. Fritter | Churro (96) | Buñuelo (2), porra (1).  TOT (1). |
| 14.10. Large fritter | Porra (58) | Churro (35), bacón (3).  DK (3), TOT (1). |
| 14.11. Millefeuille | Milhojas (46) | Pastel (22), helado (5), dulce (3), hojaldre (3), merengue (3), tarta (3), bizcocho (2), corte (2), larva (1), pastelito (1).  TOT (5), DR (3), DK (1). |
| 14.12. Zarajo* | Zarajo (21) | Entresijos (8), carne (5), madeja (4), corazón (3), hígado (1), pollo (1).  DK (38), DR (10), TOT (9). |
| 14.13. Paella | Paella (100) |  |
| 14.14. Pasty | Empanadilla (91) | Empanada (6), galleta (1).  DK (1), TOT (1). |
| 14.15. Pie | Empanada (74) | Galleta (10), torta (3), empanadilla (3), pastel (3), bizcocho (1), ensaimada (1), pizza (1), tarta (1).  TOT (3). |
| 14.16. Steak | Filete (32) | Chuleta (30), entrecot** (10), carne (9), chuletón (6), bistec** (3), cerdo frito (1), churrasco (1), costilla (1), pasta (1).  DK (6). |
| ***15. FURNITURE*** |  |  |
| 15.1. Armchair | Sillón (71) | Sofá (24), butaca (4), butacón (1). |
| 15.2. Bed | Cama (100) |  |
| 15.3. Bedside table | Mesilla (76) | Mueble (6), mesilla de noche** (5), cómoda (4), cajonera (3), mesa (1).  DR (3), DK (1), TOT (1). |
| 15.4. Bookcase | Librería (64) | Estantería (18), biblioteca (8), armario (5), vitrina (2), estante (1).  DR (1), TOT (1). |
| 15.5. Chair | Silla (100) |  |
| 15.6. Chest of drawers | Cómoda (54) | Sifonier** (11), aparador (6), consola (4), mesa (4), armario (1), cajón (1), cajonera (1), cajones (1), peinador (1), somier (1), taquillón (1), tocador (1).  DR (8), TOT (5). |
| 15.7. Couch | Diván (59) | Cheslón** (14), sillón (8), sofá (6), otomana (1), silla (1), sifonier (1), tumbona (1).  DR (5), TOT (3), DK (1). |
| 15.8. Filling cabinet | Archivador (77) | Cajón (10), fichero (4), mueble (4), armario (1), cajonera (1), cajonero (1), estantería (1).  TOT (1). |
| 15.9. Lamp | Lámpara (100) |  |
| 15.10. Lectern | Atril (31) | Caja (21), cofre (3), estuche (3), pupitre (3), ataúd (1), cajón (1), escritor (1), guarda cartas (1), madera (1), maletín (1), neceser (1), secreter (1).  DK (18), DR (8), TOT (5). |
| 15.11. Rocking chair | Mecedora (87) | Balancín (4), silla (3), hamaca (1), tumbona (1).  TOT (3), DR (1). |
| 15.12. Sofa | Sofá (81) | Tresillo (13), sillón (6). |
| 15.13. Stool | Taburete (96) | Banco (2), banqueta (1), silla (1). |
| 15.14. Table | Mesa (100) |  |
| 15.15. Wardrobe | Armario (100) |  |
|  |  |  |
| ***16. JEWELLERY*** |  |  |
| 16.1. Bangle/Bracelet | Esclava (57) | Pulsera (37), no me olvides (4), cadenita (1).  DR (1). |
| 16.2. Bracelet | Pulsera (92) | Esclava (3), cadena (2), cadenilla (1), collar (1), sortija (1). |
| 16.3. Brooch | Broche (48) | Alfiler (10), pendiente (10), colgante (3), imperdible (3), pasador (3), sortija (3), aguja (1), aguja de vestir (1), camafeo (1), joya (1), prendedor (1).  TOT (7), DK (6), DR (2). |
| 16.4. Cufflinks | Gemelos (70) | Pendiente (9), espejo (1), focos (1), mancuerna (1), sellos (1).  TOT (8), DR (5), DK (4). |
| 16.5. Diadem | Diadema (35) | Corona (48), tiara** (11), joya (1).  TOT (4), DK (1). |
| 16.6. Diamond | Diamante (77) | Cristal (4), gema** (3), brillante** (3), cuarzo (3), piedra preciosa** (3), plata (1).  DK (3), TOT (2), DR (1). |
| 16.7. Medal | Medalla (71) | Colgante (11), cadena (1), medallón (1), virgen (1).  DK (6), TOT (6), DR (3). |
| 16.8. Necklace | Collar (99) | Colgante (1). |
| 16.9. Pendant | Pendiente (95) | Adornos (2), perlas (1).  DR (2). |
| 16.10. Ring | Anillo (82) | Sortija** (17).  DR (1). |
| 16.11. Seal ring | Sello (42) | Anillo** (55), sortija (3). |
| 16.12. Tie clip | Pisa corbatas (31) | Horquilla (24), alfiler de corbata** (4), broca (4), pasador (4), imperdible (3), acero (1), alfiler (1), gemelos (1), pinza (1), presilla (1).  DK (18), DR (5), TOT (2). |
| ***17. KITCHEN UT.*** |  |  |
| 17.1. Cooking pot | Puchero (15) | Jarra (16), tetera (14), botijo (4), cacerola (4), jarrón (4), vasija (4), cafetera (3), cazo (3), cazuela (3), olla (3), calentador (1), chocolatera (1), jarro (1), marmita (1), perola (1), pota (1), tarro (1).  TOT (10), DK (6), DR (4). |
| 17.2. Cup | Taza (82) | Vaso (11), jarra (6), cuenco (1). |
| 17.3. Fondue | Fondue (75) | Recipiente (3), coctelera (1), flambeador (1), queimada (1).  DR (11), DK (4), TOT (4). |
| 17.4. Fork | Tenedor (100) |  |
| 17.5. Frying pan | Sartén (99) | Cazuela (1). |
| 17.6. Churrera* | Churrera (12) | Sacacorchos (8), grifo (5), manga (4), descorchador (3), adaptado (1), jeta (1), manguera (1), mortero (1), útil (1).  DK (59), DR (4). |
| 17.7. Peeler | Pelador (51) | Pela-patatas** (28), cúter (3), herramienta (1), lima (1), mondador** (1), monda-patatas** (1), picahielos (1), remache (1), Sacacorchos (1), utensilio (1).  DK (4), TOT (3), DR (3). |
| 17.8. Pot | Olla (69) | Cacerola (18), cazuela (8), perola (3), puchero (1).  DK (1). |
| 17.9. Saucepan | Cacerola (46) | Cazuela** (29), olla (23), puchero (1).  DR (1). |
| 17.10. Saucepan | Cazo (69) | Cacerola (19), cazuela (6), olla (3), puchero (1).  DR (1), TOT (1). |
| 17.11. Sharpening steel | Afilador (37) | Lima (18), chaira** (6), punzón (4), afila- cuchillos** (3), amolador (1), barra (1), destornillador (1), escofina (1), eslabón (1), espada (1), limón (1), picador (2).  DK (11), DR (8), TOT (4). |
| 17.12. Strainer | Colador (89) | Escurridor (5), filtro (1).  TOT (4), DR (1). |
| 17.13. Teapot | Tetera (87) | Cafetera (10), cuece leches (1).  DR (1), TOT (1). |
| ***18. MUSICAL INST.*** |  |  |
| 18.1. Accordion | Acordeón (98). | DK (1), TOT (1). |
| 18.2. Balalaika | Balalaica (13) | Guitarra (16), laúd (14), instrumento (4), sitar (3), bandurria (3), ukelele (3), cimitarra (1), citara (1), mandolín (1), órgano (1).  DK (23), DR (14), TOT (3). |
| 18.3. Bugle | Corneta (30) | Trompeta (61), trompetilla (4), saxofón (1).  DR (4). |
| 18.4. Clarinet | Clarinete (53) | Flauta (28), oboe (9), dulzaina (1), trompeta (1).  DR (4), TOT (3), DK (1). |
| 18.5. Drum | Tambor (92) | Timbal (6), bombo (2). |
| 18.6. Flute | Flauta (100) |  |
| 18.7. Guitar | Guitarra (100) |  |
| 18.8. Harmonica | Armónica (100) |  |
| 18.9. Harp | Arpa (93) | Instrumento (1), laúd (1), lira (1).  TOT (4). |
| 18.10. Maracas | Maracas (96) | Mazas (2).  DK (1), DR (1). |
| 18.11. Piano | Piano (96) | Piano de cola** (4). |
| 18.12. Saxophone | Saxofón (88) | Trompeta (9), clarinete (1).  DK (1), DR (1). |
| 18.13. Tambourine | Pandereta (100) |  |
| 18.14. Trumpet | Trompeta (97) | Musical (2), trombón (1). |
| 18.15. Tuba | Tuba (15) | Trombón (37), trompeta (22), saxofón (5), clarinete (1), corneta (1), fiscorno (1), instrumento (1), trompa (1), xilófono (1).  DK (5), DR (5), TOT (5). |
| 18.16. Violin | Violín (87) | Violonchelo (10), chelo (1), viola (1).  DR (1). |
| ***19. SPORTS/GAMES*** |  |  |
| 19.1. Soccer ball | Balón (85) | Pelota (14).  DR (1). |
| 19.2. Ball | Pelota (89) | Balón (10), bola (1). |
| 19.3. Chess | Ajedrez (100) |  |
| 19.4. Dart | Dardo (96) | Flecha (1), punzón (1).  DR (1), TOT (1). |
| 19.5. Dartboard | Diana (96) | Ruleta (1), tablero (1).  DK (1), DR (1). |
| 19.6. Diabolo | Diábolo (58) | Discóbolo (3), parábola (3), demonio (1), malabares (1), trompo (1).  TOT (13), DK (10), DR (10). |
| 19.7. Dice | Dado (100) |  |
| 19.8. Doll | Muñeca (100) |  |
| 19.9. Jump rope | Comba (66) | Saltador** (20), cuerda (10), soga (1).  DR (3). |
| 19.10. Ludo | Parchís (100) |  |
| 19.11. Racket | Raqueta (100) |  |
| 19.12. Skate | Patín (97) | Monopatín (2), patinete (1). |
| 19.13. Skis | Esquies (81) | Correas (3), pulseras (1), tirantes (1).  DK (13), DR (1). |
| 19.14. Skittle | Bolo (100) |  |
| 19.15. Spinning top | Peonza (85) | Trompo** (11), baldufa** (1), peón** (1).  DK (1), TOT (1). |
| 19.16. Table football | Futbolín (94) | Billar (5).  TOT (1). |
| ***20. TOOLS*** |  |  |
| 20.1. Axe | Hacha (97) | Azada (1), machete (1).  DR (1) |
| 20.2. Bit | Broca (74) | Taladro (4), barrena (1), berbiquí (1), hierro (1), macho (1), mecha (1), tornillo (1), tuerca (1).  DR (6), TOT (6), DK (3). |
| 20.3. Chisel | Formón (16) | Lima (20), gubia (7), lija (6), cincel (4), escoplo (3), buril (1), cuna (1), escarpelo (1), escofina (1), herramientas (1), instrumentos (1), lijadora (1), limador (1), raspador (1).  DR (15), DK (11), TOT (9). |
| 20.4. Cold chisel | Cortafríos (11) | Cincel (25), palanca (4), escoplo (3), formón (3), puntero (3), clavo (1), lima (1), palo (1), pincel (1), punta (1), punzón (1).  DK (24), DR (23), TOT (8). |
| 20.5. Hammer | Martillo (100) |  |
| 20.6. Handsaw | Serrucho (65) | Sierra (35). |
| 20.7. Leveller | Nivel (68) | Medidor (4), nivelador** (3), calibre (1), instrumento (1), regla (1).  DK (9), DR (8), TOT (5). |
| 20.8. Nail | Clavo (76) | Punta** (10), tornillo (7).  TOT (4), DR (3). |
| 20.9. Nut | Tuerca (83) | Arandela (6), tornillo (6), rosca (4), pluma (1). |
| 20.10. Pincers | Alicates (89) | Tenazas (6).  DR (4), TOT (1). |
| 20.11. Pliers | Tenazas (62) | Alicates (28), cizallas (1), llave inglesa (1).  DR (7), DK (1). |
| 20.12. Screw | Tornillo (91) | Tuerca (7).  DR (1), TOT (1). |
| 20.13. Screwdriver | Destornillador (95) | Atornillador** (4).  TOT (1). |
| 20.14. Shovel | Pala (100) |  |
| 20.15. Trowel | Llana (33) | Paleta (18), plana** (6), palustre (4), alisadora (3), aplanadora (3), espátula (3), alicatadora (1), lijadora (1), nivel (1), plancha (1).  DK (17), DR (8), TOT (1). |
| ***21. VEHICLES*** |  |  |
| 21.1. Boat | Barca (86) | Barco (9), bote (1), canoa (1), patera (1), trainera (1).  DK (1). |
| 21.2. Bus | Autobús (76) | Autocar** (23).  DK (1). |
| 21.3. Car | Coche (93) | Automóvil** (3), Peugeot 206** (2), Peugeot** (1), turismo** (1). |
| 21.4. Cart | Carro (76) | Carreta (8), carretilla (6), remolque (4), carromato** (1).  DR (3), DK (1), TOT (1). |
| 21.5. Motorbike | Moto (99) | Motocicleta** (1). |
| 21.6. Paragliding | Parapente (43) | Paracaídas (40), ala delta (8), ultraligero (1).  DR (4), TOT (3), DK (1). |
| 21.7. Plane | Avión (100) |  |
| 21.8. Scooter | Patinete (81) | Monopatín (9), patín (6), pedal (1).  DR (3). |
| 21.9. Ship | Barco (74) | Barca (15), lancha (4), yate (4), bote (1), buque** (1), maqueta (1). |
| 21.10. Skateboard | Monopatín (73) | Patinete (20), patín (5), patineta (1).  TOT (1). |
| 21.11. Tractor | Tractor (100) |  |
| 21.12. Train | Tren (93) | Cercanías** (6), metro (1). |
| 21.13. Van | Furgoneta (95) | Camioneta (4), camión (1). |
| ***22. WEAPONS*** |  |  |
| 21.1. Armour | Armadura (88) | Coraza (1), soldado (1).  TOT (7), DR (3). |
| 22.2. Arrow | Flecha (94) | Lanza (5), saeta** (1). |
| 22.3. Bayonet | Bayoneta (20) | Punzón (3), azada (1), gancho (1), lanza (1).  DK (67), DR (4), TOT (3). |
| 22.4. Boomerang | Bumerán (78) | Cerbatana (1), lombriz (1), onda (1).  TOT (9), DK (6), DR (4). |
| 22.5. Bow | Arco (100) |  |
| 22.6. Cannon | Cañón (98) | Bombarda (1).  DR (1). |
| 22.7. Crossbow | Ballesta (53) | Arco (13), cerbatana (4), herramienta (3), sierra (3), cepo (1), serrucho (1).  DK (13), DR (5), TOT (4). |
| 22.8. Grenade | Granada (90) | Bomba (5), bomba de mano** (1), cantimplora (1), mina (1).  DR (1), TOT (1). |
| 22.9. Gun | Pistola (91) | Revólver (5), máuser (1).  DK (1), DR (1), TOT (1). |
| 22.10. Helmet | Casco (79) | Sombrero (8), yelmo (3), salacot (1).  DK (6), DR (3). |
| 22.11. Machine gun | Ametralladora (52) | Metralleta (19), rifle (7), fusil (6), escopeta (4), arma (3), fusil de asalto (1).  DK (6), DR (1), TOT (1). |
| 22.12. Revolver | Revólver (39) | Pistola (61). |
| 22.13. Shield | Escudo (97) | Armazón (1), blasón (1), coraza (1). |
| 22.14. Slingshot | Tirachinas (94) | Caucho (1), gomera (1), onda (1).  TOT (3). |
| 22.15. Sword | Espada (97) | Abrecartas (2), sable (1). |
| ***23. NATURE*** |  |  |
| 23.1. Cliff | Acantilado (73) | Cabo (4), bahía (3), costa (3), montaña (3), fiordo (1), litoral (1), mar (1), paisaje (1), playa (1), precipicio (1), rocas (1).  DK (4), DR, (2) TOT (1). |
| 23.2. Cloud | Nube (100) |  |
| 23.3. Coal | Carbón (29) | Piedra (22), pasa (8), mineral (4), roca (4), ciruela (3), azabache** (1), pizarra (1).  DK (24), DR (3). |
| 23.4. Gold | Oro (30) | Nuez (6), pepita de oro** (6), mineral (5), piedra (4), roca (3), almendra (1),corcho (1), escultura (1), hongo (1), maíz (1), tótem (1), trufa (1).  DK (34), DR (5). |
| 23.5. Ice | Hielo (12) | Iceberg (54), glaciar (17), nieve (4), rocas (4), casquete (1).  DK (7), TOT (1). |
| 23.6. Iceberg | Iceberg (82) | Glaciar (14), hielo (1), mar (1), tempano (1).  TOT (1). |
| 23.7. Island | Isla (84) | Islote** (13), arco (1), arrecife (1).  TOT (1). |
| 23.8. Moon | Luna (95) | Planeta (3).  DK (1), DR (1). |
| 23.9. Mountain | Montaña (83) | Cima** (6), pico** (5), cumbre** (4), hielo (2). |
| 23.10. Puddle | Charco (28) | Mancha (4), arena (3), fósil (3), madera (3), piedra (3), suelo (3), lámina (1), manta (1), pez manta (1), raya (1), tierra (1), yeso (1).  DK (38), DR (8), TOT (1). |
| 23.11. Sea | Mar (93) | Océano** (5), agua (2). |
| 23.12. Stone | Piedra (45) | Roca** (13), cuarzo (10), mineral (6), mármol (5), alabastro (1), hueso (1), patata (1).  DK (18). |
| 23.13. Sun | Sol (75) | Luz (6), eclipse (4), destello (3), reflejo (3), resplandor (2), cristal (1), estrella (1).  TOT (3), DK (1), DR (1). |
| 23.14. Volcano | Volcán (100) |  |
| 23.15. Waterfall | Catarata (68) | Cascada** (29), mar (1), salto de agua** (1).  TOT (1). |
| 23.16. Wave | Ola (89) | Playa (6), mar (3), océano (1), oleaje** (1). |

**Note**: * = Impossible to translate into English. ** Acceptable synonym.
