# Supplementary material for: An Ecological Alternative to Snodgrass & Vanderwart: 360 High Quality Colour Images with Norms for Seven Psycholinguistic Variables
Source: PLoS One. 2012 May 25;7(5):e37527. doi: 10.1371/journal.pone.0037527 (PMC3360784; doi:10.1371/journal.pone.0037527)
Supplement: Appendix S4 — Indexes of individual item analysis including a measure of item difficulty and two indexes of item discrimination based on item-test correlations (point-biserial and biserial). (DOC) [file pone.0037527.s004.doc]

| **Item** |  | **Discrimination**  (item-total correlation) | | |
| --- | --- | --- | --- | --- |
| **English** | **Spanish** | **Difficulty** | **Point-biserial** | **Biserial** |
|  |  |  |  |  |
| ***1. ANIMALS*** |  |  |  |  |
| 1.1. Armadillo | Armadillo | .57 | .50 | .63 |
| 1.2. Bat | Murciélago | .96 | .30 | .70 |
| 1.3. Cat | Gato | 1.00 | --- | --- |
| 1.4. Cheetah | Guepardo | .32 | .07 | .09 |
| 1.5. Cow | Vaca | 1.00 | --- | --- |
| 1.6. Crocodile | Cocodrilo | .76 | .12 | .16 |
| 1.7. Dromedary | Dromedario | .50 | .23 | .29 |
| 1.8. Elephant | Elefante | 1.00 | --- | --- |
| 1.9. Giraffe | Jirafa | .97 | .15 | .40 |
| 1.10. Hippopotamus | Hipopótamo | .82 | .20 | .30 |
| 1.11. Horse | Caballo | .99 | .27 | .90 |
| 1.12. Kangaroo | Canguro | 1.00 | --- | --- |
| 1.13. Lioness | Leona | .59 | .11 | .13 |
| 1.14. Lynx | Lince | .74 | .18 | .24 |
| 1.15. Platypus | Ornitorrinco | .46 | .47 | .59 |
| 1.16. Rhino | Rinoceronte | .96 | .21 | .48 |
| 1.17. Snake | Serpiente | .92 | .28 | .51 |
| 1.18. Tapir | Tapir | .18 | .30 | .45 |
| 1.19. Tiger | Tigre | .99 | .15 | .52 |
| 1.20. Turtle | Tortuga | .90 | -.14 | -.24 |
| 1.21. Zebra | Cebra | .99 | .11 | .37 |
|  |  |  |  |  |
| ***2. BIRDS*** |  |  |  |  |
| 2.1. Barn owl | Lechuza | .47 | .16 | .20 |
| 2.2. Duck | Pato | .93 | -.09 | -.17 |
| 2.3. Goldfinch | Jilguero | .34 | .36 | .47 |
| 2.4. Goose | Oca | .52 | .35 | .43 |
| 2.5. Hen | Gallina | .87 | .27 | .43 |
| 2.6. Hummingbird | Colibrí | .71 | .40 | .53 |
| 2.7. Kiwi | Kiwi | .32 | .41 | .54 |
| 2.8. Magpie | Urraca | .21 | .38 | .53 |
| 2.9. Ostrich | Avestruz | .96 | .28 | .65 |
| 2.10. Owl | Búho | .81 | .03 | .04 |
| 2.11. Partridge | Perdiz | .51 | .60 | .75 |
| 2.12. Pelican | Pelícano | .70 | .42 | .55 |
| 2.13. Penguin | Pingüino | .99 | -.07 | -.22 |
| 2.14. Pheasant | Faisán | .26 | .40 | .54 |
| 2.15. Pigeon | Paloma | .99 | .29 | .98 |
| 2.15. Pigeon | Paloma | .99 | .29 | .98 |
| 2.16. Raven | Cuervo | .77 | .19 | .27 |
| 2.17. Rooster | Gallo | .90 | .24 | .41 |
| 2.18. Seagull | Gaviota | .83 | .30 | .44 |
| 2.19. Sparrow | Gorrión | .50 | .35 | .44 |
| 2.20. Toucan | Tucán | .58 | .18 | .23 |
|  |  |  |  |  |
| ***3. BODY PARTS*** |  |  |  |  |
| 3.1. Arm | Brazo | .96 | -.03 | -.06 |
| 3.2. Beard | Barba | .43 | .13 | .16 |
| 3.3. Bone | Hueso | 1.00 | --- | --- |
| 3.4. Brain | Cerebro | .86 | -.25 | -.40 |
| 3.5. Ear | Oreja | .99 | -.06 | -.19 |
| 3.6. Eye | Ojo | 1.00 | --- | --- |
| 3.7. Finger | Dedo | .90 | -.05 | -.09 |
| 3.8. Foot | Pie | 1.00 | --- | --- |
| 3.9. Hand | Mano | 1.00 | --- | --- |
| 3.10. Kidney | Riñón | .47 | .10 | .12 |
| 3.11. Leg | Pierna | 1.00 | --- | --- |
| 3.12. Liver | Hígado | .68 | .31 | .40 |
| 3.13. Lung | Pulmón | .42 | .09 | .11 |
| 3.14. Mouth | Boca | .24 | -.07 | -.09 |
| 3.15. Nose | Nariz | 1.00 | --- | --- |
| 3.16. Pelvis | Pelvis | .26 | .09 | .12 |
| 3.17. Skull | Cráneo | .43 | .07 | .09 |
| 3.18. Toe | Uña | .95 | -.02 | -.04 |
| 3.19. Tongue | Lengua | .92 | .17 | .31 |
| 3.20. Vertebra | Vértebra | .34 | .33 | .43 |
|  |  |  |  |  |
| ***4. FLOWERS*** |  |  |  |  |
| 4.1. Araceae | Cala | .23 | .23 | .32 |
| 4.2. Bellflowers | Campanillas | .16 | .21 | .31 |
| 4.3. Carnation | Clavel | .85 | .35 | .53 |
| 4.4. Daisy | Margarita | .95 | .01 | .01 |
| 4.5. Geranium | Geranio | .18 | .24 | .36 |
| 4.6. Lilac | Lilas | .26 | .22 | .29 |
| 4.7. Orchid | Orquídea | .36 | .48 | .61 |
| 4.8. Pansy | Pensamiento | .30 | .31 | .41 |
| 4.9. Poppy | Amapola | .89 | .18 | .30 |
| 4.10. Rose | Rosa | .98 | -.11 | -.29 |
| 4.11. Sunflower | Girasol | .56 | .04 | .05 |
| 4.12. Tulip | Tulipán | .61 | .28 | .35 |
|  |  |  |  |  |
| ***5. FRUITS*** |  |  |  |  |
| 5.1. Apple | Manzana | .99 | -.10 | -.34 |
| 5.2. Avocado | Aguacate | .70 | .36 | .47 |
| 5.3. Banana | Plátano | .98 | .26 | .70 |
| 5.4. Cherries | Cerezas | .95 | .11 | .24 |
| 5.5. Coconut | Coco | .75 | .15 | .21 |
| 5.6. Custard apple | Chirimoya | .51 | .49 | .62 |
| 5.6. Custard apple | Chirimoya | .51 | .49 | .62 |
| 5.7. Fig | Higo | .80 | .33 | .47 |
| 5.8. Flat peach | Paraguaya | .41 | .46 | .58 |
| 5.9. Grapes | Uvas | .96 | -.11 | -.25 |
| 5.10. Kiwi fruit | Kiwi | .96 | -.04 | -.08 |
| 5.11. Lemon | Limón | .90 | .06 | .11 |
| 5.12. Mango | Mango | .27 | .18 | .24 |
| 5.13. Melon | Melón | 1.00 | --- | --- |
| 5.14. Orange | Naranja | 1.00 | --- | --- |
| 5.15. Peach | Melocotón | .89 | .23 | .38 |
| 5.16. Pear | Pera | 1.00 | --- | --- |
| 5.17. Pomegranate | Granada | .84 | .30 | .44 |
| 5.18. Quince | Membrillo | .41 | .13 | .17 |
| 5.19. Redcurrant | Grosella | .14 | .24 | .38 |
| 5.20. Strawberry | Fresa | .94 | -.14 | -.29 |
| 5.21. Watermelon | Sandía | .97 | .27 | .69 |
|  |  |  |  |  |
| ***6. INSECTS*** |  |  |  |  |
| 6.1. Ant | Hormiga | .96 | .52 | 1.20 |
| 6.2. Bee | Abeja | .61 | -.02 | -.02 |
| 6.3. Beetle | Escarabajo | .82 | .12 | .17 |
| 6.4. Butterfly | Mariposa | .99 | .18 | .63 |
| 6.5. Centipede | Ciempiés | .83 | .16 | .24 |
| 6.6. Cockroach | Cucaracha | .48 | .05 | .07 |
| 6.7. Dragonfly | Libélula | .46 | .26 | .33 |
| 6.8. Fly | Mosca | .99 | .12 | .41 |
| 6.9. Grasshopper | Saltamontes | .76 | .33 | .45 |
| 6.10. Ladybird | Mariquita | .95 | .34 | .72 |
| 6.11. Mosquito | Mosquito | .81 | .42 | .60 |
| 6.12. Moth | Polilla | .67 | .50 | .64 |
| 6.13. Praying mantis | Mantis | .68 | .17 | .22 |
| 6.14. Scorpion | Escorpión | .84 | .31 | .46 |
| 6.15. Spider | Araña | .79 | .02 | .04 |
| 6.16. Termite | Termita | .27 | .01 | .01 |
| 6.17. Wasp | Avispa | .88 | .34 | .55 |
|  |  |  |  |  |
| ***7. M.CREATURES*** |  |  |  |  |
| 7.1. Cockle | Berberecho | .16 | .20 | .30 |
| 7.2. Crab | Cangrejo | .93 | .03 | .05 |
| 7.3. Dolphin | Delfín | .99 | .13 | .43 |
| 7.4. Eel | Anguila | .18 | .16 | .23 |
| 7.5. Goose barnacle | Percebe | .67 | .34 | .44 |
| 7.6. Killer whale | Orca | .55 | .38 | .48 |
| 7.7. Lobster | Bogavante | .19 | .24 | .35 |
| 7.8. Manatee | Manatí | .08 | .29 | .54 |
| 7.9. Mussel | Mejillón | .92 | .32 | .59 |
| 7.10. Narwhal | Narval | .03 | .16 | .41 |
| 7.11. Oyster | Ostra | .38 | .47 | .60 |
| 7.12. Pomfret | Palometa | .11 | .34 | .56 |
| 7.12. Pomfret | Palometa | .11 | .34 | .56 |
| 7.13. Ray | Raya | .54 | .31 | .39 |
| 7.14. Razor-shell | Navaja | .78 | .44 | .62 |
| 7.15. Shark | Tiburón | .89 | .31 | .51 |
| 7.16. Sperm whale | Cachalote | .24 | .27 | .37 |
| 7.17. Starfish | Estrella de mar | 1.00 | --- | --- |
| 7.18. Whale | Ballena | .44 | .31 | .39 |
|  |  |  |  |  |
| ***8. NUTS*** |  |  |  |  |
| 8.1. Acorn | Bellota | .71 | .19 | .25 |
| 8.2. Almond | Almendra | .63 | .33 | .43 |
| 8.3. Chestnut | Castaña | .72 | .46 | .62 |
| 8.4. Date | Dátil | .40 | .22 | .28 |
| 8.5. Hazelnut | Avellana | .61 | .33 | .42 |
| 8.6. Peanut | Cacahuete | .92 | .02 | .04 |
| 8.7. Pine kernel | Piñón | .08 | .20 | .37 |
| 8.8. Pipe | Pipa | .82 | .00 | -.01 |
| 8.9. Pistachio | Pistacho | .76 | .22 | .30 |
| 8.10. Raisin | Pasa | .83 | .38 | .56 |
| 8.11. Walnut | Nuez | .97 | .10 | .25 |
|  |  |  |  |  |
| ***9. TREES*** |  |  |  |  |
| 9.1. Black poplar | Chopo | .01 | .11 | .39 |
| 9.2. Cedar | Cedro | .01 | .10 | .34 |
| 9.3. Cypress | Ciprés | .55 | .62 | .78 |
| 9.4. Eucalyptus | Eucalipto | .08 | .17 | .32 |
| 9.5. Fig tree | Higuera | .01 | .11 | .38 |
| 9.6. Fir | Abeto | .39 | .25 | .32 |
| 9.7. Holm oak | Encina | .31 | .46 | .60 |
| 9.8. Olive tree | Olivo | .53 | .24 | .30 |
| 9.9. Palm tree | Palmera | .97 | .50 | 1.32 |
| 9.10. Pine tree | Pino | .58 | .38 | .48 |
| 9.11. Willow | Sauce | .66 | .35 | .46 |
|  |  |  |  |  |
| ***10. VEGETABLES*** |  |  |  |  |
| 10.1. Artichoke | Alcachofa | .79 | .33 | .47 |
| 10.2. Asparagus | Espárrago | .85 | .47 | .72 |
| 10.3. Cabbage | Repollo | .28 | .22 | .29 |
| 10.4. Carrot | Zanahoria | 1.00 | --- | --- |
| 10.5. Cauliflower | Coliflor | .85 | .03 | .04 |
| 10.6. Celery | Apio | .61 | .24 | .30 |
| 10.7. Chard | Acelgas | .72 | .20 | .27 |
| 10.8. Cucumber | Pepino | .97 | .12 | .33 |
| 10.9. Eggplant | Berenjena | .93 | .09 | .18 |
| 10.10. Endive | Escarola | .54 | .36 | .45 |
| 10.11. Leek | Puerro | .65 | .27 | .35 |
| 10.12. Lettuce | Lechuga | .97 | .05 | .13 |
| 10.13. Onion | Cebolla | .99 | -.06 | -.21 |
| 10.14. Pepper | Pimiento | .99 | .03 | .10 |
| 10.15. Potato | Patata | 1.00 | --- | --- |
| 10.15. Potato | Patata | 1.00 | --- | --- |
| 10.16. Pumpkin | Calabaza | .83 | .33 | .49 |
| 10.17. Red cabbage | Lombarda | .33 | .38 | .50 |
| 10.18. Spinach | Espinaca | .46 | .25 | .32 |
| 10.19. Tomato | Tomate | 1.00 | --- | --- |
| 10.20. Turnip | Nabo | .08 | .33 | .59 |
|  |  |  |  |  |
| ***11. BUILDINGS*** |  |  |  |  |
| 11.1. Castle | Castillo | .96 | .10 | .22 |
| 11.2. Cathedral | Catedral | .81 | .18 | .26 |
| 11.3. Church | Iglesia | .85 | .02 | .03 |
| 11.4. Factory | Fábrica | .63 | .20 | .26 |
| 11.5. Granary | Hórreo | .51 | .46 | .58 |
| 11.6. House | Casa | .93 | .08 | .15 |
| 11.7. Lighthouse | Faro | .96 | .19 | .43 |
| 11.8. Mill | Molino | .97 | .06 | .16 |
| 11.9. Pagoda | Pagoda | .26 | .29 | .39 |
| 11.10. Palace | Palacio | .33 | .19 | .25 |
| 11.11. Pyramid | Pirámide | .84 | -.01 | -.01 |
| 11.12. Shanty | Chabola | .79 | .28 | .39 |
| 11.13. Silo | Silo | .29 | .24 | .31 |
| 11.14. Skyscraper | Rascacielos | .73 | .03 | .04 |
| 11.15. Tower | Torre | .78 | .01 | .02 |
|  |  |  |  |  |
| ***12. CLOTHING*** |  |  |  |  |
| 12.1. Bathrobe | Albornoz | .91 | -.08 | -.14 |
| 12.2. Biretta | Birrete | .36 | .11 | .14 |
| 12.3. Cap | Gorra | .96 | .05 | .11 |
| 12.4. Clog | Zueco | .75 | .28 | .39 |
| 12.5. Coat | Abrigo | .96 | .23 | .52 |
| 12.6. Glove | Guante | 1.00 | --- | --- |
| 12.7. Jacket | Chaqueta | .70 | .10 | .14 |
| 12.8. Shirt | Camisa | .99 | .08 | .27 |
| 12.9. Shoe | Zapato | 1.00 | --- | --- |
| 12.10. Skirt | Falda | .99 | .03 | .11 |
| 12.11. Socks | Calcetín | 1.00 | --- | --- |
| 12.12. Trousers | Pantalón | 1.00 | --- | --- |
| 12.13. Undershirt | Camiseta | .99 | .17 | .59 |
|  |  |  |  |  |
| ***13. DESK MATERIAL*** |  |  |  |  |
| 13.1. Compasses | Compás | .97 | .45 | 1.19 |
| 13.2. Eraser | Borrador | .04 | .16 | .37 |
| 13.3. Felt-tip pen | Rotulador | .93 | .20 | .37 |
| 13.4. Folder | Carpeta | .96 | -.14 | -.32 |
| 13.5. Fountain pen | Pluma | .88 | -.05 | -.07 |
| 13.6. Ink pad | Tampón | .28 | .45 | .60 |
| 13.7. Paperclip | Clip | .99 | -.11 | -.36 |
| 13.8. Pen | Bolígrafo | 1.00 | --- | --- |
| 13.9. Pencil | Lápiz | .99 | -.19 | -.64 |
| 13.10. Pencil sharpener | Sacapuntas | .97 | -.01 | -.02 |
| 13.10. Pencil sharpener | Sacapuntas | .97 | -.01 | -.02 |
| 13.11. Ruler | Regla | .99 | .41 | 1.38 |
| 13.12. Set square | Cartabón | .54 | .27 | .34 |
| 13.13. Square | Escuadra | .64 | .06 | .08 |
| 13.14. Rubber stamp | Sello | .58 | .21 | .26 |
| 13.15. Stapler | Grapadora | .93 | .33 | .61 |
|  |  |  |  |  |
| ***14. FOOD*** |  |  |  |  |
| 14.1. Anchovy | Anchoas | .92 | .44 | .81 |
| 14.2. Black pudding | Morcilla | .56 | .26 | .33 |
| 14.3. Caviar | Caviar | .58 | .00 | .01 |
| 14.4. Cheese | Queso | 1.00 | --- | --- |
| 14.5. Chorizo | Chorizo | .94 | .08 | .16 |
| 14.6. Cookie | Galleta | .93 | .22 | .42 |
| 14.7. Crème caramel | Flan | .96 | .36 | .84 |
| 14.8. Fritter | Churro | .96 | .25 | .59 |
| 14.9. Large fritter | Porra | .58 | .48 | .61 |
| 14.10. Millefeuille | Milhojas | .46 | .26 | .33 |
| 14.11. NT (Zarajo) | Zarajo | .21 | .43 | .61 |
| 14.12. Paella | Paella | 1.00 | --- | --- |
| 14.13. Pasty | Empanadilla | .90 | -.09 | -.16 |
| 14.14. Pie | Empanada | .73 | .15 | .20 |
| 14.15. Steak | Filete | .31 | .13 | .17 |
|  |  |  |  |  |
| ***15. FURNITURE*** |  |  |  |  |
| 15.1. Armchair | Sillón | .71 | .13 | .17 |
| 15.2. Bed | Cama | 1.00 | --- | --- |
| 15.3. Bedside table | Mesilla | .76 | -.01 | -.02 |
| 15.4. Bookcase | Librería | .64 | .13 | .16 |
| 15.5. Chair | Silla | 1.00 | --- | --- |
| 15.6. Chest of drawers | Cómoda | .53 | .08 | .10 |
| 15.7. Couch | Diván | .59 | .14 | .18 |
| 15.8. Filling cabinet | Archivador | .76 | .31 | .43 |
| 15.9. Lamp | Lámpara | 1.00 | --- | --- |
| 15.10. Lectern | Atril | .29 | .26 | .34 |
| 15.11. Rocking chair | Mecedora | .86 | .26 | .40 |
| 15.12. Sofa | Sofá | .81 | -.22 | -.31 |
| 15.13. Stool | Taburete | .96 | .25 | .56 |
| 15.14. Table | Mesa | 1.00 | --- | --- |
| 15.15. Wardrobe | Armario | 1.00 | --- | --- |
|  |  |  |  |  |
| ***16. JEWELLERY*** |  |  |  |  |
| 16.1. Bangle/Bracelet | Esclava | .57 | .10 | .13 |
| 16.2. Bracelet | Pulsera | .92 | .33 | .60 |
| 16.3. Brooch | Broche | .47 | .05 | .06 |
| 16.4. Cufflinks | Gemelos | .70 | .38 | .50 |
| 16.5. Diadem | Diadema | .34 | .06 | .08 |
| 16.6. Diamond | Diamante | .78 | .18 | .25 |
| 16.7. Medal | Medalla | .71 | .16 | .21 |
| 16.8. Necklace | Collar | .99 | .03 | .10 |
| 16.8. Necklace | Collar | .99 | .03 | .10 |
| 16.9. Pendant | Pendiente | .95 | .17 | .36 |
| 16.10. Ring | Anillo | .82 | -.03 | -.04 |
| 16.11. Seal ring | Sello | .42 | .00 | .00 |
| 16.12. Tie clip | Pisa corbatas | .31 | .17 | .22 |
|  |  |  |  |  |
| ***17. KITCHEN UTENSILES*** |  |  |  |  |
| 17.1. Cooking pot | Puchero | .14 | .30 | .47 |
| 17.2. Cup | Taza | .82 | .36 | .53 |
| 17.3. Fondue | Fondue | .74 | .34 | .46 |
| 17.4. Fork | Tenedor | 1.00 | --- | --- |
| 17.5. Frying pan | Sartén | .99 | .25 | .86 |
| 17.6. NT (Churrera) | Churrera | .11 | .28 | .46 |
| 17.7. Peeler | Pelador | .49 | .16 | .20 |
| 17.8. Pot | Olla | .69 | -.18 | -.23 |
| 17.9. Saucepan | Cacerola | .46 | .03 | .03 |
| 17.10. Small saucepan | Cazo | .68 | .21 | .28 |
| 17.11. Sharpening steel | Afilador | .39 | .14 | .18 |
| 17.12. Strainer | Colador | .89 | .19 | .31 |
| 17.13. Teapot | Tetera | .86 | .25 | .40 |
|  |  |  |  |  |
| ***18. MUSICAL INSTRUMENTS*** |  |  |  |  |
| 18.1. Accordion | Acordeón | .97 | .03 | .09 |
| 18.2. Balalaika | Balalaica | .13 | .19 | .31 |
| 18.3. Bugle | Corneta | .30 | .49 | .65 |
| 18.4. Clarinet | Clarinete | .53 | .29 | .37 |
| 18.5. Drum | Tambor | .92 | .03 | .06 |
| 18.6. Flute | Flauta | 1.00 | --- | --- |
| 18.7. Guitar | Guitarra | 1.00 | --- | --- |
| 18.8. Harmonica | Armónica | .92 | .36 | .66 |
| 18.9. Harp | Arpa | .92 | .25 | .46 |
| 18.10. Maracas | Maracas | .96 | .02 | .05 |
| 18.11. Piano | Piano | .96 | .07 | .16 |
| 18.12. Saxophone | Saxofón | .87 | .18 | .29 |
| 18.13. Tambourine | Pandereta | .99 | .41 | 1.38 |
| 18.14. Trumpet | Trompeta | .97 | .13 | .32 |
| 18.15. Tuba | Tuba | .14 | .17 | .26 |
| 18.16. Violin | Violín | .86 | .03 | .04 |
|  |  |  |  |  |
| ***19. SPORTS/GAMES*** |  |  |  |  |
| 19.1. Soccer ball | Balón | .85 | .25 | .39 |
| 19.2. Ball | Pelota | .89 | .02 | .03 |
| 19.3. Chess | Ajedrez | 1.00 | --- | --- |
| 19.4. Dart | Dardo | .95 | .18 | .37 |
| 19.5. Dartboard | Diana | .95 | .25 | .54 |
| 19.6. Diabolo | Diábolo | .59 | .43 | .54 |
| 19.7. Dice | Dado | 1.00 | --- | --- |
| 19.8. Doll | Muñeca | 1.00 | --- | --- |
| 19.9. Jump rope | Comba | .65 | -.03 | -.04 |
| 19.9. Jump rope | Comba | .65 | -.03 | -.04 |
| 19.10. Ludo | Parchís | .99 | -.20 | -.64 |
| 19.11. Racket | Raqueta | 1.00 | --- | --- |
| 19.12. Skate | Patín | .97 | .27 | .72 |
| 19.13. Ski | Esquís | .81 | .05 | .08 |
| 19.14. Skittle | Bolo | .95 | .25 | .53 |
| 19.15. Spinning top | Peonza | .84 | .15 | .23 |
| 19.16. Table football | Futbolín | .93 | .10 | .19 |
|  |  |  |  |  |
| ***20. TOOLS*** |  |  |  |  |
| 20.1. Axe | Hacha | .96 | .26 | .61 |
| 20.2. Bit | Broca | .73 | .44 | .59 |
| 20.3. Chisel | Formón | .15 | .32 | .49 |
| 20.4. Cold chisel | Cortafríos | .13 | .23 | .37 |
| 20.5. Hammer | Martillo | 1.00 | --- | --- |
| 20.6. Handsaw | Serrucho | .65 | .16 | .21 |
| 20.7. Leveller | Nivel | .68 | .49 | .64 |
| 20.8. Nail | Clavo | .76 | .19 | .26 |
| 20.9. Nut | Tuerca | .81 | .01 | .01 |
| 20.10. Pincers | Alicates | .89 | .33 | .55 |
| 20.11. Pliers | Tenazas | .63 | .25 | .32 |
| 20.12. Screw | Tornillo | .90 | .08 | .15 |
| 20.13. Screwdriver | Destornillador | .94 | .18 | .37 |
| 20.14. Shovel | Pala | .96 | .40 | .90 |
| 20.15. Trowel | Llana | .32 | .57 | .74 |
|  |  |  |  |  |
| ***21. VEHICLES*** |  |  |  |  |
| 21.1. Boat | Barca | .86 | .30 | .47 |
| 21.2. Bus | Autobús | .76 | -.25 | -.35 |
| 21.3. Car | Coche | .93 | .06 | .12 |
| 21.4. Cart | Carro | .75 | .36 | .50 |
| 21.5. Motorbike | Moto | .99 | .13 | .44 |
| 21.6. Paragliding | Parapente | .43 | .32 | .41 |
| 21.7. Plane | Avión | 1.00 | --- | --- |
| 21.8. Scooter | Patinete | .81 | .11 | .16 |
| 21.9. Ship | Barco | .74 | .03 | .05 |
| 21.10. Skateboard | Monopatín | .73 | .35 | .47 |
| 21.11. Tractor | Tractor | .99 | -.20 | -.64 |
| 21.12. Train | Tren | .93 | .08 | .15 |
| 21.13. Van | Furgoneta | .95 | -.01 | -.03 |
|  |  |  |  |  |
| ***22. WEAPONS*** |  |  |  |  |
| 22.1. Armour | Armadura | .88 | .13 | .21 |
| 22.2. Arrow | Flecha | .94 | .28 | .57 |
| 22.3. Bayonet | Bayoneta | .20 | .30 | .43 |
| 22.4. Boomerang | Bumerán | .77 | .21 | .29 |
| 22.5. Bow | Arco | .99 | .13 | .44 |
| 22.6. Cannon | Cañón | .98 | .07 | .18 |
| 22.7. Crossbow | Ballesta | .53 | .41 | .52 |
| 22.8. Grenade | Granada | .89 | .15 | .24 |
| 22.8. Grenade | Granada | .89 | .15 | .24 |
| 22.9. Gun | Pistola | .90 | .18 | .31 |
| 22.10. Helmet | Casco | .79 | .24 | .34 |
| 22.11. Machine gun | Ametralladora | .51 | .08 | .10 |
| 22.12. Revolver | Revólver | .39 | .17 | .21 |
| 22.13. Shield | Escudo | .96 | .11 | .24 |
| 22.14. Slingshot | Tirachinas | .95 | .27 | .56 |
| 22.15. Sword | Espada | .97 | -.11 | -.27 |
|  |  |  |  |  |
| ***23. NATURE*** |  |  |  |  |
| 23.1. Cliff | Acantilado | .69 | .04 | .06 |
| 23.2. Cloud | Nube | 1.00 | --- | --- |
| 23.3. Coal | Carbón | .29 | .09 | .12 |
| 23.4. Gold | Oro | .29 | .02 | .03 |
| 23.5. Ice | Hielo | .11 | .15 | .25 |
| 23.6. Iceberg | Iceberg | .81 | .29 | .43 |
| 23.7. Island | Isla | .84 | .08 | .12 |
| 23.8. Moon | Luna | .95 | -.04 | -.07 |
| 23.9. Mountain | Montaña | .83 | -.26 | -.38 |
| 23.10. Puddle | Charco | .26 | .16 | .22 |
| 23.11. Sea | Mar | .93 | .03 | .05 |
| 23.12. Stone | Piedra | .44 | .12 | .14 |
| 23.13. Sun | Sol | .75 | -.05 | -.06 |
| 23.14. Volcano | Volcán | .99 | -.11 | -.39 |
| 23.15. Waterfall | Catarata | .67 | .24 | .31 |
| 23.16. Wave | Ola | .89 | .05 | .08 |

**Note:** NT = No translation into English.
